# First levantine fossil murines shed new light on the earliest intercontinental dispersal of mice

**DOI:** 10.1038/s41598-019-47894-y

**Published:** 2019-08-29

**Authors:** Raquel López-Antoñanzas, Sabrina Renaud, Pablo Peláez-Campomanes, Dany Azar, George Kachacha, Fabien Knoll

**Affiliations:** 1 Laboratoire de Paléontologie, Institut des Sciences de l’Évolution (UMR-CNRS 5554), Université de Montpellier, Montpellier, France; 2 Departamento de Paleobiología, Museo Nacional de Ciencias Naturales-CSIC, Madrid, Spain; 3 Laboratoire de Biométrie et Biologie Evolutive (UMR-CNRS 5558), Université Lyon 1, Villeurbanne, France; 4 Natural Sciences Department, Faculty of Sciences II, Lebanese University, Fanar, Lebanon; 5 ARAID—Fundación Conjunto Paleontológico de Teruel-Dinopolis, Teruel, Spain; 6 Department of Earth and Environmental Sciences, School of Natural Science, University of Manchester, Manchester, United Kingdom

**Keywords:** Palaeontology, Palaeontology

## Abstract

Recent extensive field prospecting conducted in the Upper Miocene of Lebanon resulted in the discovery of several new fossiliferous localities. One of these, situated in the Zahleh area (Bekaa Valley, central Lebanon) has yielded a particularly diverse vertebrate fauna. Micromammals constitute an important part of this assemblage because not only do they represent the first Neogene rodents and insectivores from Lebanon, but they are also the only ones from the early Late Miocene of the Arabian Peninsula and circumambient areas. Analyses of the murines from Zahleh reveal that they belong to a small-sized early *Progonomys*, which cannot be assigned to any of the species of the genus hitherto described. They are, thereby, shown to represent a new species: *Progonomys manolo*. Morphometric analyses of the outline of the first upper molars of this species suggest a generalist and omnivorous diet. This record sheds new light onto a major phenomenon in the evolutionary history of rodents, which is the earliest dispersal of mice. It suggests that the arrival of murines in Africa got under way through the Levant rather than *via* southern Europe and was monitored by the ecological requirements of *Progonomys*.

## Introduction

The Murinae represent the largest subfamily of mammals, comprising 656 species and 135 genera^1^. This group of rodents constitutes one of the most evolutionarily successful clades of mammals, displaying an outstanding diversity, a virtually cosmopolitan distribution and a wide array of feeding preferences. They supposedly originated in the Middle Miocene of southern Asia, as their earliest representatives (*Antemus*) have been recorded in Siwaliks deposits about 14 Ma of age^2–4^. However, the full development of the dental pattern seen in modern murines (with three strong chevrons in the M1) was achieved later, with the appearance of *Progonomys* in Late Miocene deposits of the same area dating 12.4 Ma^2–4^. *Progonomys* is probably one of the most important taxa in the history of murine rodents, not only because it was the earliest taxon to acquire the derived dental characters of the crown murines, but also because it was the first modern representative of the group to spread out of southern Asia. *Progonomys* dispersed from southern Asia to Europe and Africa at the beginning of the Late Miocene (~11 Ma). This dispersal and that of the three-toed horse *Hipparion* are seen as the two most remarkable Late Miocene events in the history of Old World mammals^5^. The possible routes and timing of this first murine dispersal have been the subject of hypotheses^6–8^, which have remained educated guesses in the absence of data from the Arabian area. Fossil material collected during excavations in the summer of 2013 and 2018 in the Upper Miocene of Lebanon provides an opportunity to address this gap in our knowledge. Our fieldworks resulted indeed in the discovery of the first *Progonomys* from the whole Arabian area, which constitutes the first physical evidence about the dispersal of the earliest murines from southern Asia to Africa through the Levant.

## Results

### Systematic palaeontology

Rodentia Bowdich, 1821

Muridae Illiger, 1811

Murinae Illiger, 1811

*Progonomys* Schaub, 1938

Synonymy: *Sinapodemus* Sen, 2003

**Figure 1:**
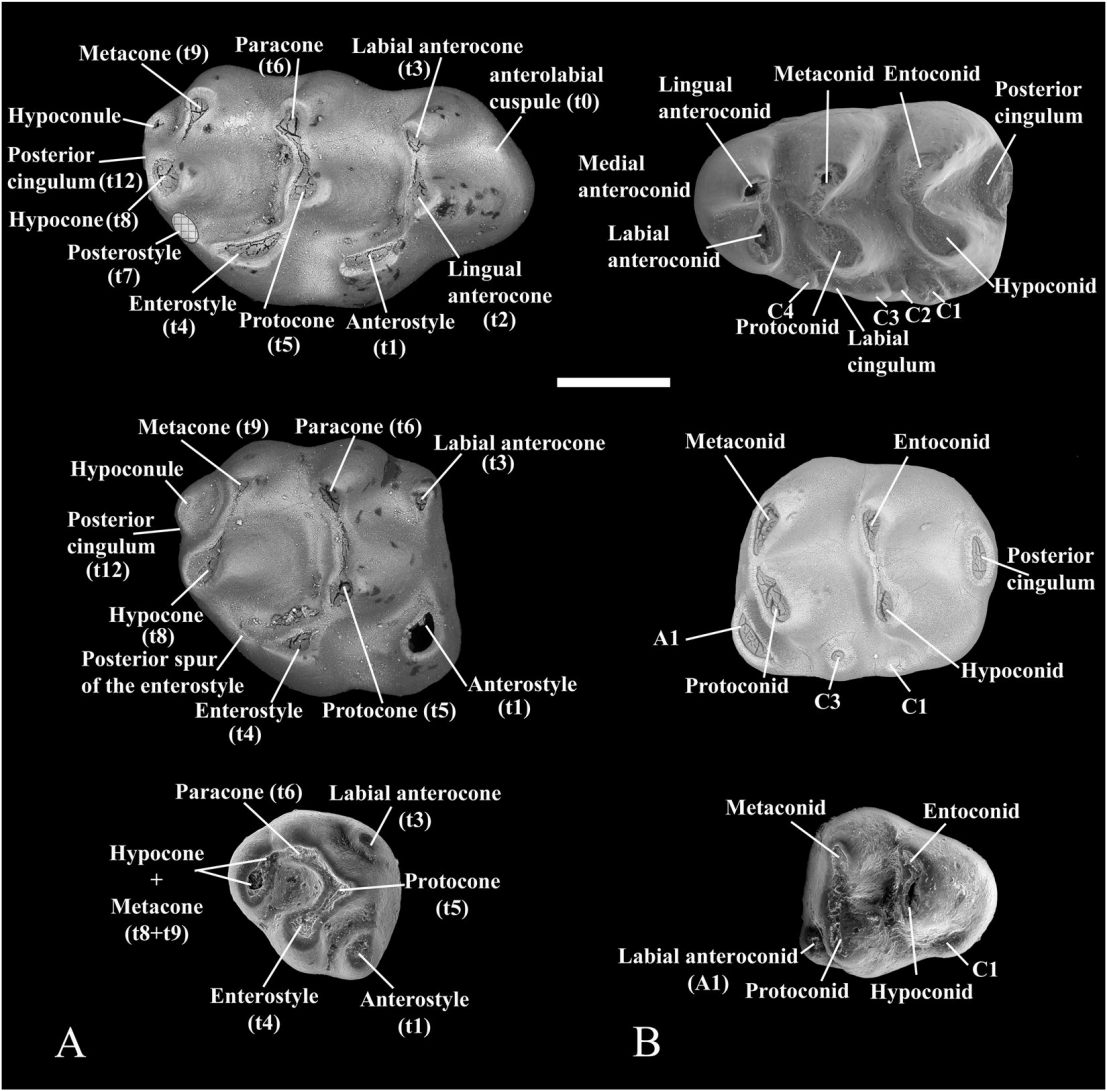
Dental murine terminology used in this work. (**A**) Upper molars (M1-M3 from top to bottom); (**B**) Lower molars (m1–m3 from top to bottom). Scale bar equals 500 µ.

**Figure 2:**
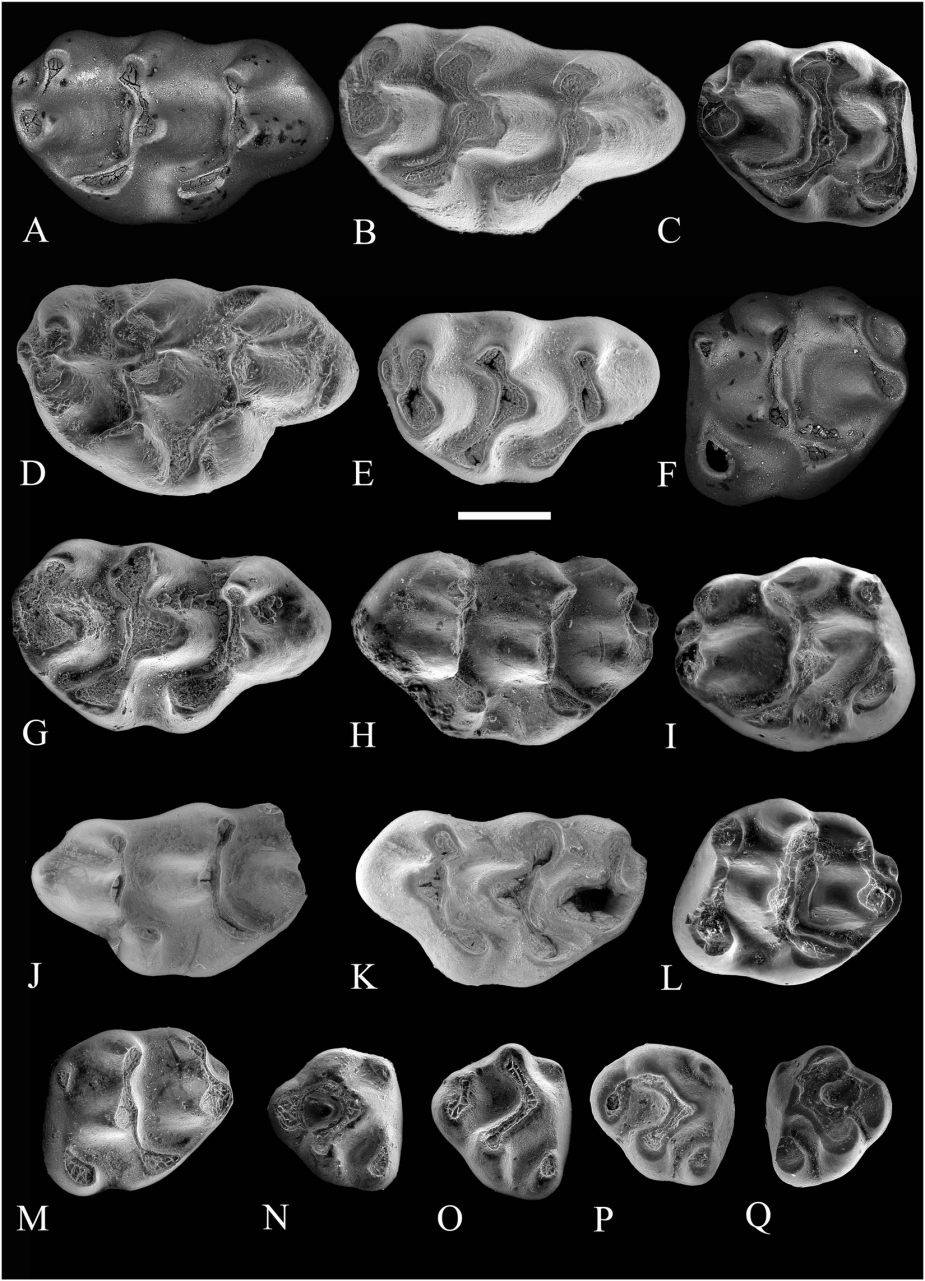
Occlusal views of upper cheek teeth of *Progonomys manolo* sp. nov. (**A**) Zahleh 49, right M1; **(B)** Zahleh 134, right M1; **(C)** Zahleh 37, right M2; **(D)** Zahleh 15, right M1; **(E)** Zahleh 127, right M1; **(F)** Zahleh 48, left M2; **(G)** Zahleh 95, right M1; **(H)** Zahleh 94, left M1; **(I)** Zahleh 88, right M2; **(J)** Zahleh 90, left M1; **(K)** Zahleh 83, left M1; **(L)** Zahleh 96, left M2; **(M)** Zahleh 97, left M2; **(N)** Zahleh 72, right M3; **(O)** Zahleh 69, right M3; **(P)** Zahleh 34, right M3; **(Q)** Zahleh 16, left M3. Images obtained from Scanning Electron Microscopy (SEM). Scale bar equals 500 µ.

**Figure 3:**
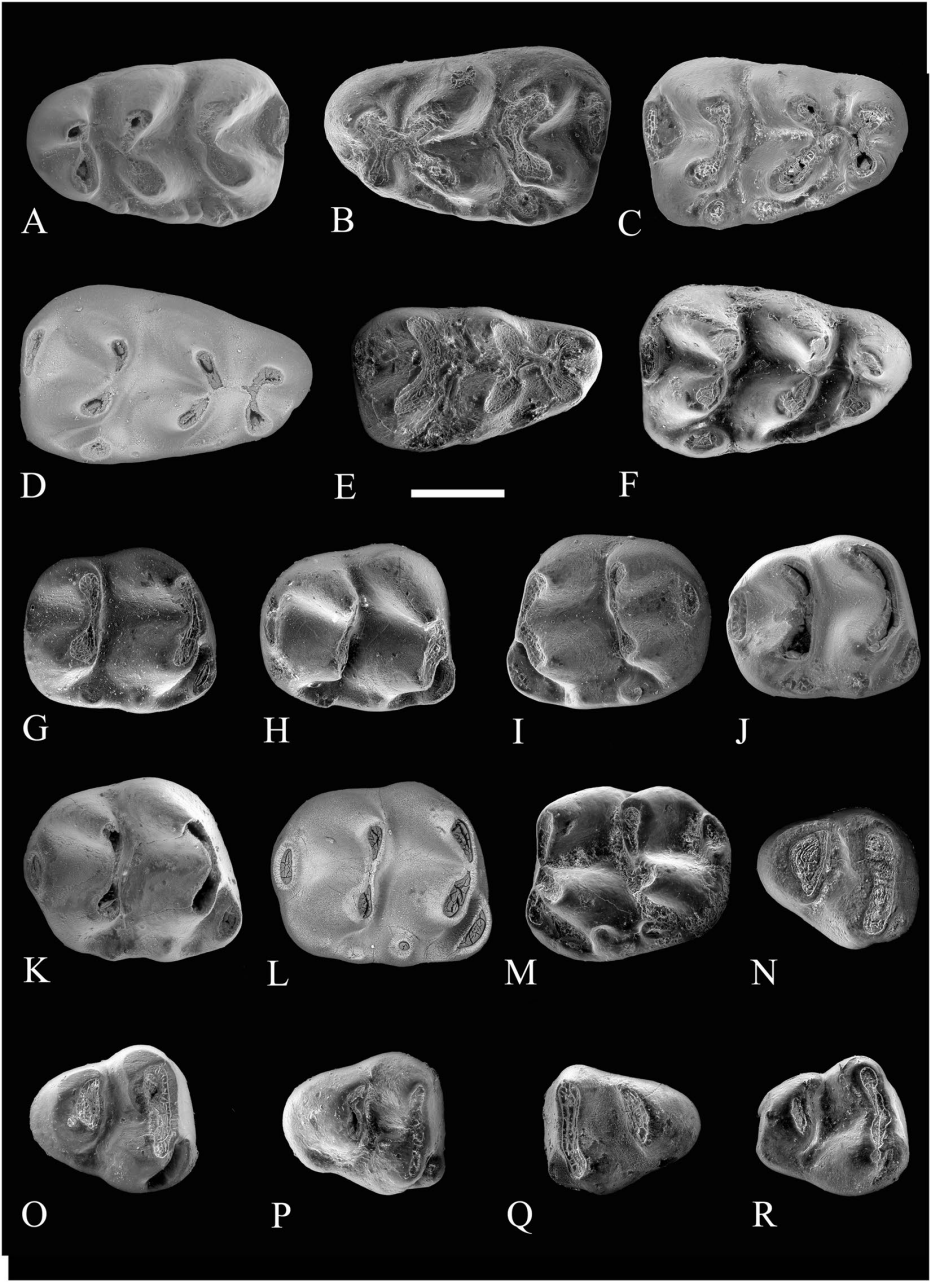
Occlusal views of lower cheek teeth of *Progonomys manolo* sp. nov. (**A)** Zahleh 125, left m1; **(B)** Zahleh 8, left m1; **(C)** Zahleh 1, right m1; **(D)** Zahleh 51, right m1; **(E)** Zahleh 79, right m1; **(F)** Zahleh 91, right m1; **(G)** Zahleh 129, right m2; **(H)** Zahleh 65, right m2; **(I)** Zahleh 128, left m2; **(J)** Zahleh 72, right m2; **(K)** Zahleh 64, right m2; **(L)** Zahleh 44, right m2; **(M)** Zahleh 99, left m2; **(N)** Zahleh 62, right m3; **(O)** Zahleh 86, right m3; **(P)** Zahleh 85, right m3; **(Q)** Zahleh 71, left m3; **(R)** Zahleh 68, right m3. Images obtained from Scanning Electron Microscopy (SEM). Scale bar equals 500 µ.

*Progonomys manolo* sp. nov. (Figs 1–3)

#### Etymology

In loving memory of Manuel López Gálvez. Manolo is a hypocorism of Manuel. The noun is used in apposition.

#### Holotype

Right first upper molar (M1) (Zahleh 49) (Fig. 2A and Supplementary Fig. S1, which is available via the online Supplementary Content). This and the paratype specimens are provisionally housed in the University of Montpellier, Montpellier, France. They will be stored in the Museum of Natural History of the Lebanese University in Fanar (Lebanon) upon completion of their study.

#### Paratypes

See online Supplementary Content (Supplementary Text S1).

#### Locality and horizon

Wadi Al Aarayech, Zahleh, Lebanon. All the specimens come from the same gastropod-rich layer in the lower part of the informally named “Zahleh Formation”^9^ (for the precise stratigraphic context see López-Antoñanzas *et al*.^10^). Late Miocene (probably equivalent to MN9 in terms of MN zonation).

#### Diagnosis

Primitive species of *Progonomys* with small cheek teeth; M1 with slender occlusal outline, with oval and anteroposteriorly elongated t1, t1 bis absent, t1 and t4 noticeably lower than t2 and t5 and posterior to them, t12 short but distinct; in labial view M1 shows a large and posteriorly inclined t9, which is parallel to t6; M2 and M3 with large t1; m1 without medial anterior cuspid (tma), very weak or absent anteroconid-metaconid connexion and C1 isolated from the hypoconid; m2 with rather straight chevrons, particularly the posterior one; all lower molars with weak labial cingulum but quite developed cingulum cuspids.

#### Differential diagnosis

*Progonomys manolo* is unmistakably smaller than *Progonomys sinensis*, *Progonomys clauzoni*, *Progonomys woelferi* and *Progonomys cathalai*, which are younger species of the genus. The Lebanese taxon is of about the same size as the oldest species belonging to this genus (the ancient populations of *Progonomys hispanicus, Progonomys hussaini*, *Progonomys minus*, *Progonomys ibrahimi*, *Progonomys morganae* and *Progonomys shalaensis*) and the younger *Progonomys debruijni*. Some of the latter taxa are slightly smaller (*Progonomys debruijni*, *Progonomys morganae, Progonomys shalaensis*) or larger (*Progonomys hussaini*) than *Progonomys manolo*. *Progonomys manolo* differs from *Progonomys hussaini* in having t1 more strongly connected to t2, weaker longitudinal connections between the anteroconid and the first lobe on m1 and more developed cingulum cuspids. *Progonomys manolo* is different from *Progonomys morganae* in having a short and cusp-like t12 on M1, in having a large cusp t1 on M2 and M3 and in having cingulum cuspids on m1 that are more numerous and developed than in the Pakistani species. *Progonomys manolo* is distinct from *Progonomys debruijni* in having a weaker connection between the anteroconid and the first lobe on m1, a shorter and less ridge-like t12 on the upper molars, less compressed and posteriorly situated t1, and a usually higher number of cingulum cuspids on m2. The Lebanese taxon differs from *Progonomys minus* in having M1 with more slender and elongated occlusal outline (see below) and cusp t4 situated in a more posterior position, in having an interrupted labial cingulum that bears at least three cingulum cuspids on m1 and in having cusps t1 and t5 not connected to each other on M3. *Progonomys manolo* differs from *Progonomys hispanicus* in having lower and more labiolingually compressed lingual cusps on M1 with t1 more elongated and posteriorly placed and t12 better developed. The anteroconid is, on m1 of *Progonomys manolo*, more anteriorly located (leaving no place for the development of a tma) than in *Progonomys hispanicus*, the lingual cuspids are less developed in *Progonomys manolo* than in *Progonomys hispanicus* and cingulum cuspid C1 is isolated in *Progonomys manolo*, whereas it seems to be connected to the hypoconid in *Progonomys hispanicus*. Cingulum cuspid C1 is less developed on m2 of the Lebanese taxon than in *Progonomys hispanicus*.

#### Description

M1–All cusps are well differentiated and are not transversely aligned; cusps t1 and t4 are noticeably lower than t2 and t5 and cusps t3, t6 and t9 lack any longitudinal connection between them. Some specimens show a small t0 (anterolabial cusp) and some of them (e.g. Zahleh 134, Zahleh 90, Fig. 2B,J) also have a precingulum but none of them show t1 bis. Cusps t2 and t3 are completely aligned, whereas t1 is located well posterior to them. This latter is labiolingually compressed and weakly connected to t2. Cusp t2 is very large and situated near the midline of the tooth. In contrast, cusp t3 is much smaller. Cusp t4 is placed posterior to the t5–t6 complex. It is connected to cusp t8 by a thin ridge. The lingual cusps t1 and t4 are oval and longitudinally elongated. None of the specimens have a lingual cingulum between cusps t1 and t4 but some of them (e.g. Zahleh 15, Zahleh 94, Fig. 2D,H and Supplementary Fig. S1) have a tiny accessory cusp (neoenterostyle) at this place. The labial cusps t6 and t9 are well-developed and of about the same size; they are parallel in lateral view and always separated one from another. Cusp t6 usually shows a very short but distinct posterior paracone spur. Cusp t9 is not anterior to t8 and the t8-t9 complex forms practically a right angle with the longitudinal axis of the tooth. Cusp t8 is considerably higher than the remaining cusps. Cusp t7 is always absent. Cusps t9 and t12 are separated by a narrow sinus in all but the heavily worn specimens (e.g. Zahleh 95, Fig. 2G), in which t12 has virtually disappeared. The roots are not preserved in any of the specimens.

Specimen Zahleh 02 (Supplementary Fig. S2) is discarded from the sample of specimens considered as belonging to the new Lebanese taxon (see below).

M2–The morphology of M2 resembles that of M1. These teeth lack longitudinal connections between labial cusps. Cusps t1 and t3 are always present. Cusp t1 is very large and t3, even though it is more variable in size, is smaller than t1 but well-developed in all specimens. Most of the specimens (7 out of 11) show these two cusps connected by a ridge (e.g. Zahleh 37, Zahleh 88, Zahleh 96 Fig. 2C,I,L and Supplementary Fig. S1). Cusp t4 is large and longitudinally elongated, posterior to the t5-t6 complex. These three cusps (t4, t5, t6), on one side, and t9 and t8, on the other, are well connected to one another. Cusp t4 is connected to t8 by a low ridge (posterior spur of the enterostyle, *sensu* Kimura *et al*., 2017). These teeth have neither t7 nor small accessory cusps. All specimens show a cusp-like t12, which is separated from t9 by a small and narrow sinus.

M3–These teeth are triangular in occlusal outline. Cusps t1 and t3 are always present and they are not connected to each other. Cusp t1 is large, whereas t3, even though distinct in all specimens, is the smallest cusp and is situated as high as or higher than t1. However, a few specimens (Zahleh 16 Fig. 2Q and Zahleh 138) have a tiny t3 that is located lower than t1, near the base of the tooth. The largest cusps of the teeth are t4, t5 and t8. The first chevron, which is constituted by cusps t4-t5-t6, forms a right angle. Cusps t8 and t9 are usually fused. However, cusp t9 is still recognizable in some specimens (e.g. Zahleh 63, Zahleh 69, see Fig. 2O). In all specimens but Zahleh 34 and Zahleh 69 (Fig. 2O and Supplementary Fig. S1), cusp t4 connects to t8. No roots are preserved in any of the M3 found.

m1–The prelobe is composed of two cuspids of similar size, the labial and lingual anteroconids, which are transversely aligned and connected to each other. They are situated centrally on the anterior part of the molar. The anteroconid and the metaconid are not connected except in worn specimens. In this case, the connexion is made between the metaconid and the lingual anteroconid (e.g. Zahleh 01, Zahleh 43, Zahleh 79, Fig. 3B,C,E). The tma (medial anterior cuspid) is absent. The protoconid and metaconid, as well as the hypoconid and metaconid, are transversely connected. Both complexes are similar in size. All specimens lack the longitudinal crest as well as the posterior spur of the metaconid and the anterior spur of the entoconid. As a result, there is a continuous central sinusid from the labial side of the m1 to the lingual side. All specimens show at least three cingulum cuspids (C1, C3 and C4). C1 is the largest one. C2 is much less developed and is absent in some specimens (e.g. Zahleh 01, Fig. 3C). C3 is always distinct but less developed than C1. C4 is usually well developed, but may be embedded in the labial cingulum (e.g. Zahleh 51, Zahleh 91, see Fig. 3D,F). All specimens show a weak labial cingulum. The posterior cingulum is large and isolated. It is located slightly lingual to the longitudinal axis of the teeth. These teeth are two-rooted.

m2–The occlusal outline of these teeth is rounded-rectangular. The anterolabial cuspid (A1) is prominent. In some specimens (e.g. Zahleh 99, Fig. 3M), the anterolabial cuspid A1 is small but an additional and well-developed cuspid A1’ (*sensu* Wessels^6^) is present. The second chevron (entoconid-hypoconid complex) is fairly straight and the cuspids rather narrow. All specimens have a large accessory anterior cingulum cuspid (C3) and a smaller but well-developed posterior cingulum cuspid (C1). C3 is in contact with the protoconid, whereas C1 is isolated. In addition, a few specimens (e.g. Zahleh 44, Zahleh 66, Fig. 3L and Supplementary Fig. S1) also have an additional labial cingulum cuspid (C2). The posterolophid is large and cusp-like. These specimens are two-rooted.

m3–These teeth are triangular in occlusal outline with the maximal length on the lingual side. The anterolabial cuspid (A1) is variable in size, from large (e.g. Zahleh 68, Zahleh 85, Zahleh 86, Fig. 3O,R and Supplementary Fig. S1) to medium (Zahleh 62, Zahleh 71, Fig. 3Q), but is always distinct. The protoconid and the metaconid are well connected forming a single, long and straight first chevron. The second chevron is constituted by the fusion of the entoconid and the hypoconid. In the less worn specimens (e.g. Zahleh 85, Fig. 3P), a constriction between these two cuspids allows to differentiate the entoconid from the hypoconid. A posterolabial cingulum cuspid (C1 *sensu* Wessels, 2009) is visible in some specimens (e.g. Zahleh 67, Zahleh 85, Zahleh 86, Fig. 3O,P). These teeth are two-rooted.

#### Comparisons

A detailed comparison between *Progonomys manolo*, all known species of *Progonomys* and other murines is available as online Supplementary Content (Supplementary Text S1).

### Morphometrics

**Figure 4:**
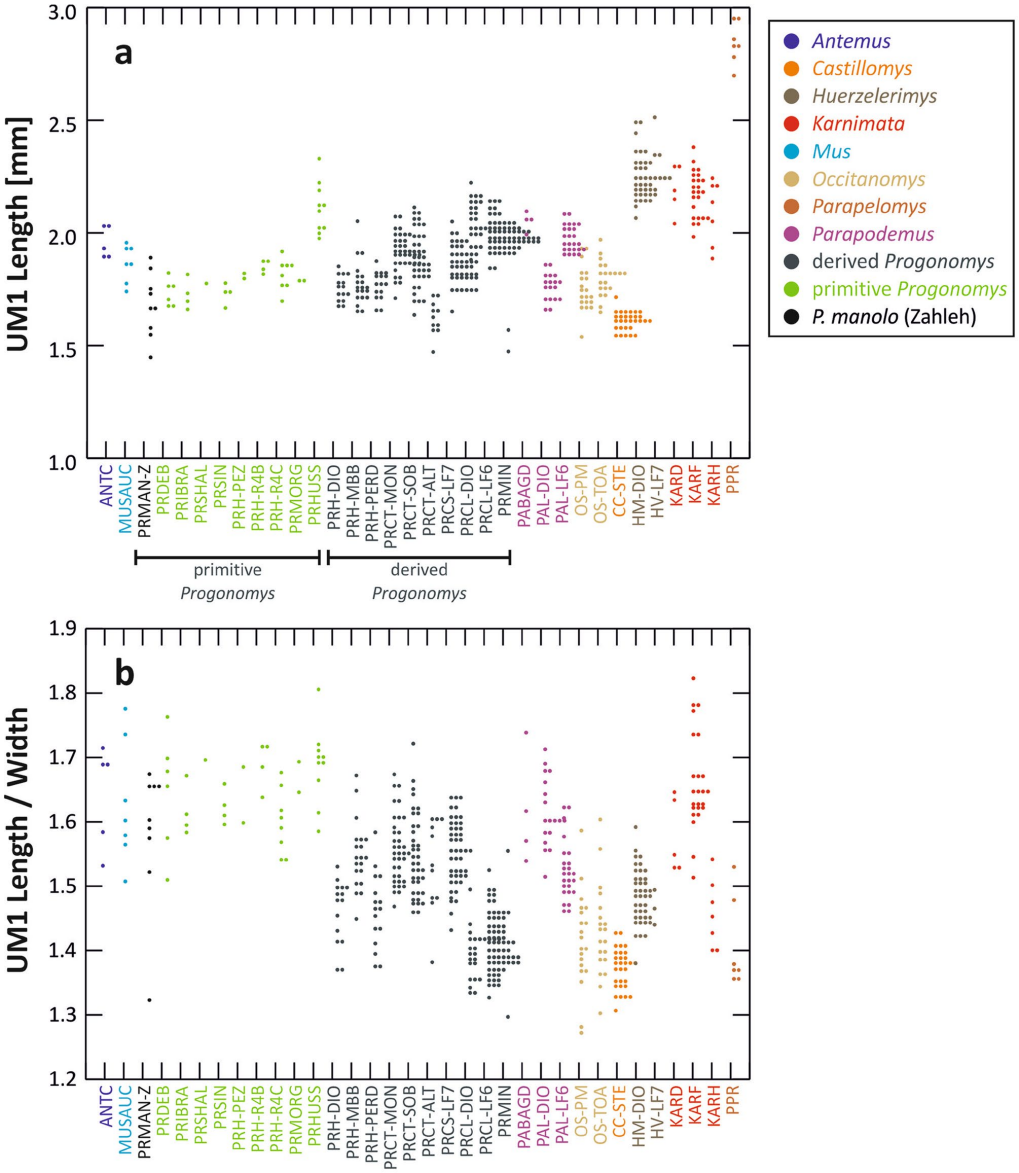
Size and elongation of the first upper molar from the total dataset and *Progonomys manolo*. (**a**) Tooth maximal length; (**b**) Tooth length over width ratio. The groups along the horizontal axis correspond to species per deposits. For codes, see Supplementary Table S3. The group “Primitive *Progonomys*” includes specimens of *Progonomys debruijni, Progonomys ibrahimi, Progonomys shalaensis, Progonomys sinensis, Progonomys hussaini, Progonomys morganae* and early *Progonomys hispanicus* (R4B, R4C and Pezinok) in green and of *Progonomys manolo* in black.

The teeth of the Lebanese species are amongst the shortest of the dataset (Fig. 4a). For the majority, they are within the range of variation of early *Progonomys* and *Mus auctor*.

The length/width ratio provided a measure of the elongation of the tooth (Fig. 4b). The teeth from Zahleh appear heterogeneous in this respect. Most teeth share a high length/width ratio, indicating elongate teeth. Such elongation has also been found in early *Progonomys*, as well as in *Mus* and *Antemus*. In contrast, one specimen (Zahleh 02, Supplementary Fig. S2) displays a very low length/width ratio, corresponding to a broad tooth, such as those of *Castillomys, Occitanomys* and *Progonomys clauzoni*.

**Figure 5:**
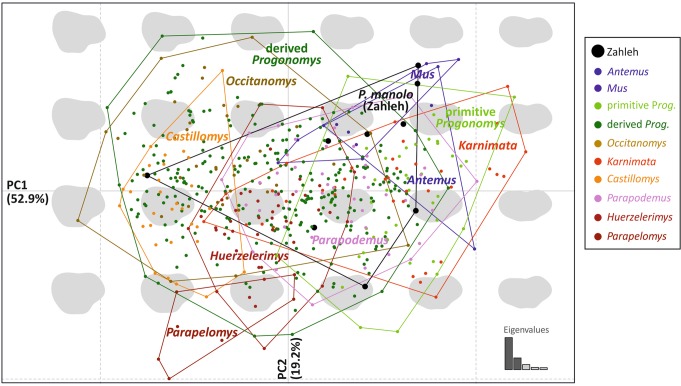
Tooth shape variation in the morphological space corresponding to the first and second axes of a PCA on the Fourier coefficients. Each dot corresponds to a tooth. Convex hulls enclose the range of variation of each genus in the dataset. Teeth from Zahleh are plotted within this range of variation (large black dots). Reconstructions of outlines visualizing the shape variation along the axes are superimposed onto the morphological space. The group “Primitive *Progonomys*” includes specimens of *Progonomys debruijni, Progonomys ibrahimi, Progonomys shalaensis, Progonomys sinensis, Progonomys hussaini, Progonomys morganae* and early *Progonomys hispanicus* (R4B, R4C and Pezinok) in green and of *Progonomys manolo* in black.

Regarding the molar shape, the analysis of the total data set (541 teeth) shows an important variation in shape, even within a single genus (Fig. 5). The first PC axis (52.9% of the total variance) opposes elongate teeth to broad teeth. The second PC axis (19.2%) tends to oppose teeth with a large posterior part, especially on the labial side, to outline with a narrow forepart and discrete lingual cusps. Most of the teeth from Zahleh cluster towards positive PC1 scores, corresponding to narrow teeth, such as those belonging to *Antemus*, *Mus* and early *Progonomys*. Nonetheless, one tooth (Zahleh 02, Supplementary Fig. S2) is located towards extreme negative scores and, therefore, corresponds to a very broad tooth.

In size and shape, the Zahleh sample may appear quite variable. Discarding Zahleh 02, the coefficient of variation of the M1 length, the variance of the ratio M1 length/width and the shape variance (sum of the variance of the series of FCs) were estimated on the original sample and bootstrap estimates, and compared to similar original and bootstrapped estimates in other *Progonomys* samples as well as the sample of *Mus auctor*, and in three samples of modern *Apodemus sylvaticus* for which genetic data can definitely discard the issue of interspecific mixing. The Zahleh sample displayed a variation in length and shape within the upper range of what is observed in other samples, but other fossil samples provided similar high variation in M1 length (*Progonomys cathalai* from Soblay) and shape (*Progonomys hispanicus* from Masia del Barbo) (Supplementary Fig. S3). The variation of the length/width ratio of the Zahleh sample was low and in the range of modern wood mice populations. So, we consider the sample from Zahleh (except for Zahleh 02) as belonging to a single species.

**Figure 6:**
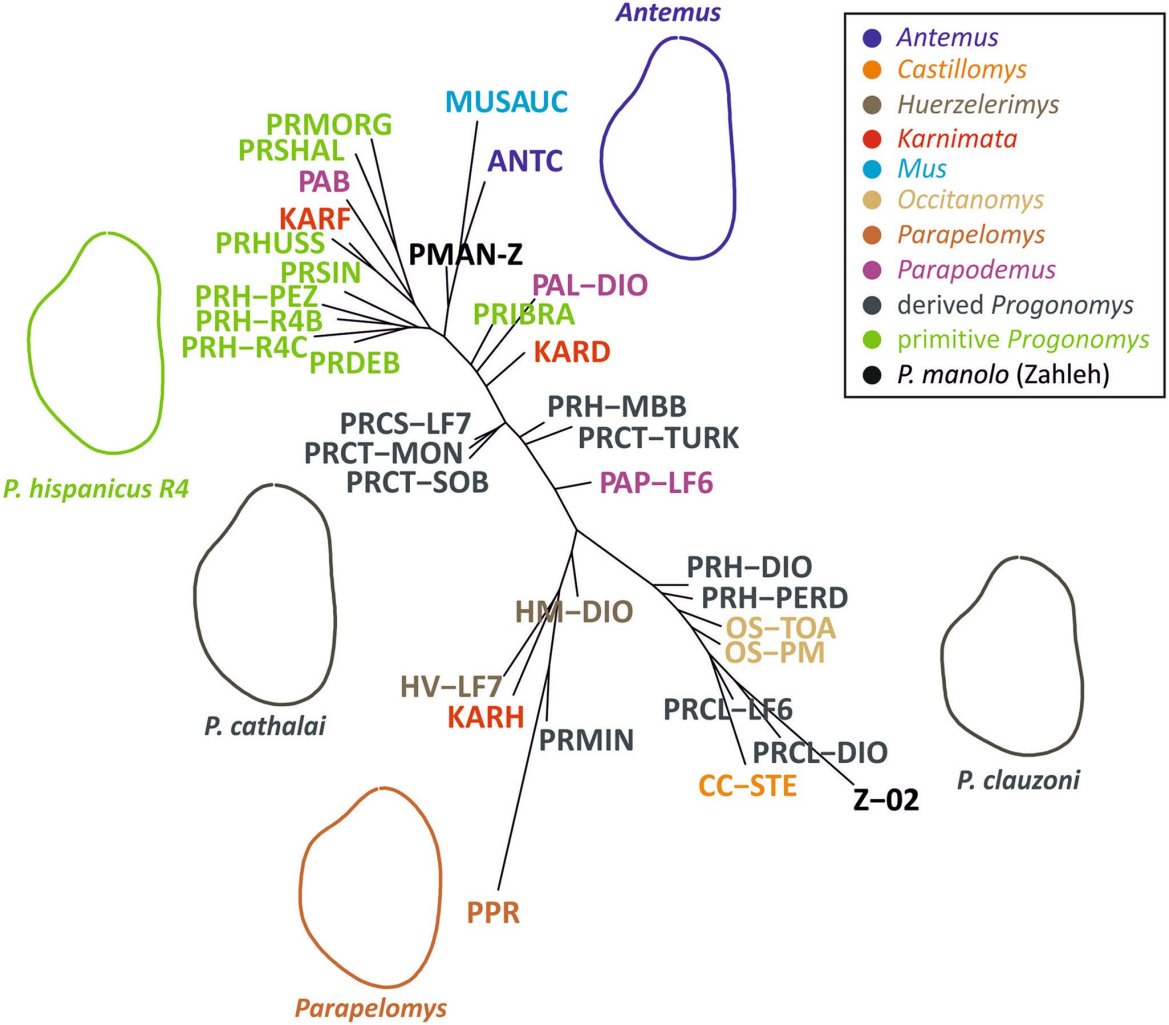
Relationships between groups (species per deposits), represented by a neighbor joining tree on the group means of the 14 Fourier coefficients. Zahleh 02 has been considered apart from the rest of the specimens. The outline of some group means or genera are provided to illustrate the corresponding shape variation. The group “Primitive *Progonomys*” includes specimens of *Progonomys debruijni, Progonomys ibrahimi, Progonomys shalaensis, Progonomys sinensis, Progonomys hussaini, Progonomys morganae* and early *Progonomys hispanicus* (R4B, R4C and Pezinok) in green and *Progonomys manolo* in black.

A neighbor-joining tree on the Euclidean distances between group means (species per deposits) based on the 14 shape variables provides further visualization of the relationships, discarding within-group variation but including all dimensions of shape differences (Fig. 6). Due to the differences found in terms of size, elongation and shape between Zahleh 02 and the other Lebanese teeth, the sample from Zahleh has been split into Zahleh 02 (Supplementary Fig. S2) and the rest of the specimens. The groups are arranged along a gradient of narrow to broad outlines. Primitive *Progonomys* correspond to such extreme elongations and cluster close to *Antemus* and *Mus auctor*. *Progonomys manolo* from Zahleh (exclusive of Zahleh 02) clusters with this group of samples. *Parapodemus*, *Progonomys cathalai* and *Karnimata darwini* are scattered in intermediate positions between the endmembers opposing elongated to broad outlines. Finally, two groups of broad outlines diverge. The first one is constituted by *Huerzelerimys, Progonomys minus, Karnimata huxleyi* and *Parapelomys*. This group is characterized by very round outlines with a reduced forepart. The second group comprises the younger *Progonomys hispanicus, Progonomys clauzoni, Occitanomys* and *Castillomys*, all sharing broad outlines with a prominent forepart. Tooth Zahleh 02 clusters with this group of samples.

Based on these analyses, *Progonomys manolo* is nested within primitive murines. Hence, its univariate measurements (length, width and length/width) were compared to four groups of primitive murines with relatively good sample sizes (Supplementary Table S4). *Progonomys manolo* was significantly smaller than early *Progonomys hispanicus* and *Progonomys hussaini*. However, regarding its length/width ratio, it only differed slightly from *Progonomys hussaini*. Because of the limited sample sizes, no multivariate comparison was performed.

### Evolution of the genus

The main characters usually considered to establish the evolutionary stage of *Progonomys* species are the occlusal outline of M1, the development of additional cusp t1 bis on M1 and longitudinal connections between cusps, particularly t6 and t9 on upper molars, the acquisition of a tma on m1 and the presence of a longitudinal spur on m1 and m2^11^. Additional important characters are the position, height and outline (in occlusal view) of cusps t1 and t4 on M1, the development of the labial cingulum and cingulum cuspids on lower molars as well as the longitudinal connections of the anteroconid with the metaconid-protoconid on m1.

Wessels^6^ synonymized the species *Progonomys castilloae*, *Progonomys hussaini*, *Progonomys minus* and *Progonomys sinensis* with *Progonomys cathalai*. However, the differences in size (Fig. 4 and Supplementary Fig. S4), morphology and occlusal outline (see morphometric analysis above) between *Progonomys cathalai*, *Progonomys sinensis*, *Progonomys minus* and *Progonomys hussaini* falsify this hypothesis. Likewise, some of the oldest *Progonomys* assemblages from Algeria (e.g. Bou Hanifia 2 and 5 and Oued Zra^12,13^) and Turkey (Altıntaş and Kutahya^6^ and Tuḡlu^14^) considered as *Progonomys cathalai*, probably belong to different species (see supplementary Text S1). *Progonomys ibrahimi* is an additional taxon of *Progonomys*, formerly considered as representing a distinct genus, *Sinapodemus*^15^ (supplementary Text S1), that has been recorded from the Late Miocene of Turkey.

*Progonomys manolo* from Lebanon is a small primitive *Progonomys* characterized by elongate M1 in occlusal outline, low and elongate cusps t1 and t4 that are weakly connected with t2 and t5, respectively, cusp t1 very posteriorly situated, no t1 bis and no longitudinal connections between the labial cusps but with lingual cusp t4 connected to t8. Concerning the lower molars, m1 lacks tma and is characterized by a weak labial cingulum with quite developed cingulum cuspids, of which C1 remains isolated from the hypoconid. All or most of these characters are present in early *Progonomys* (*Progonomys hussaini, Progonomys ibrahimi*, *Progonomys debruijni, “Progonomys cathalai”* from Turkey and early populations of *Progonomys hispanicus*).

*Progonomys hussaini* is known from several localities (JAL-101, Y 311, Y 450 and Y 259) of the Nagri Formation (Potwar Plateau, Pakistan) with an estimated age of 10.5–10.1 Ma^4^, whereas the younger *Progonomys debruijni* has been found at localities Y 182 and Y 367 of the Dhok Pathan Formation (Potwar Plateau, northern Pakistan), dating 9.2–9 Ma^4,16^. The oldest *Progonomys* from Turkey has been recorded from the Tuḡlu Formation, *circa* 11–10.5 Ma^14^. Other ancient Turkish populations of *Progonomys* are those from Altıntaş and Kutahya^14^ and *Progonomys ibrahimi* from Sinap Tepe^15^ (*circa* 9.9 Ma). The oldest populations of *Progonomys hispanicus* have been found at several localities in Spain (Cortasogas 2B, Belmonte, Pedregueras 2C, Masía de la Roma 4B and 4C)^11^, Austria (Richardhof–Wald and Neusiedl am See)^17^ and Slovakia (Pezinok)^18^, with estimated ages ranging from 9.98 (Spain) to 9.7 Ma (Austria and Slovakia). Given that (1) the evolutionary stage of the Lebanese taxon is comparable to these primitive populations of *Progonomys*, particularly to the oldest ones (*Progonomys hussaini*, *Progonomys ibrahimi*) and (2) it stands to reason that the arrival of *Progonomys* in Levant took place before the taxon reached the Iberian Peninsula several thousand kilometres to the West, the deposition of the “Zahleh Formation” could have been initiated prior to 9.98 Ma (MN9/MN10 boundary). This is consistent with a preliminary analysis of the association of micromammals from the “Zahleh Formation” that we uncovered. Indeed, we found together with *Progonomys manolo* various molars of the cricetodontini *Byzantinia* that show an evolutionary stage between that of *Byzantinia ozansoyi* and that of *Byzantinia nikosi* from the Late Miocene sites of Bayraktepe I (MN7 + 8) and Bayraktepe II (MN9), respectively^19^. Interestingly, the teeth of the Lebanese *Byzantinia* closely recall those found at the locality TU 19 from the Tuḡlu Formation (Çankiri Basin, central Anatolia) that were identified as *Byzantinia* sp. by Joniak and de Bruijn^14^ (pl I, 14 and 15). This locality has been considered as early Late Miocene and correlated with European Mammal Neogene unit MN9. These data are consistent with the hypothesis of López-Antoñanzas *et al*.^10^ who, after studying the ctenodactyline from the “Zahleh Formation”, concluded that its evolutionary stage is similar to that of the gundi from the Egyptian deposits of Sheik Abdahalla (11–10 Ma). López-Antoñanzas *et al*.^10^ inferred for the lacustrine deposits of Zahleh (the lower part at least) an age several million years older, one stage more ancient (Tortonian rather than Messinian), than previously suggested^20,21^. The present work allows narrowing down the estimated age of the “Zahleh Formation”: it may have started to be deposited during the early Tortonian. Given the proximity of Lebanon to the African continent (the shortest distance on land between Zahleh and Port Said is about 500 km), *Progonomys manolo* might have given rise to the populations of *Progonomys* that settled in Africa and, thereby, been at the root of the successful dispersal phenomena that ensued.

## Discussion

**Figure 7:**
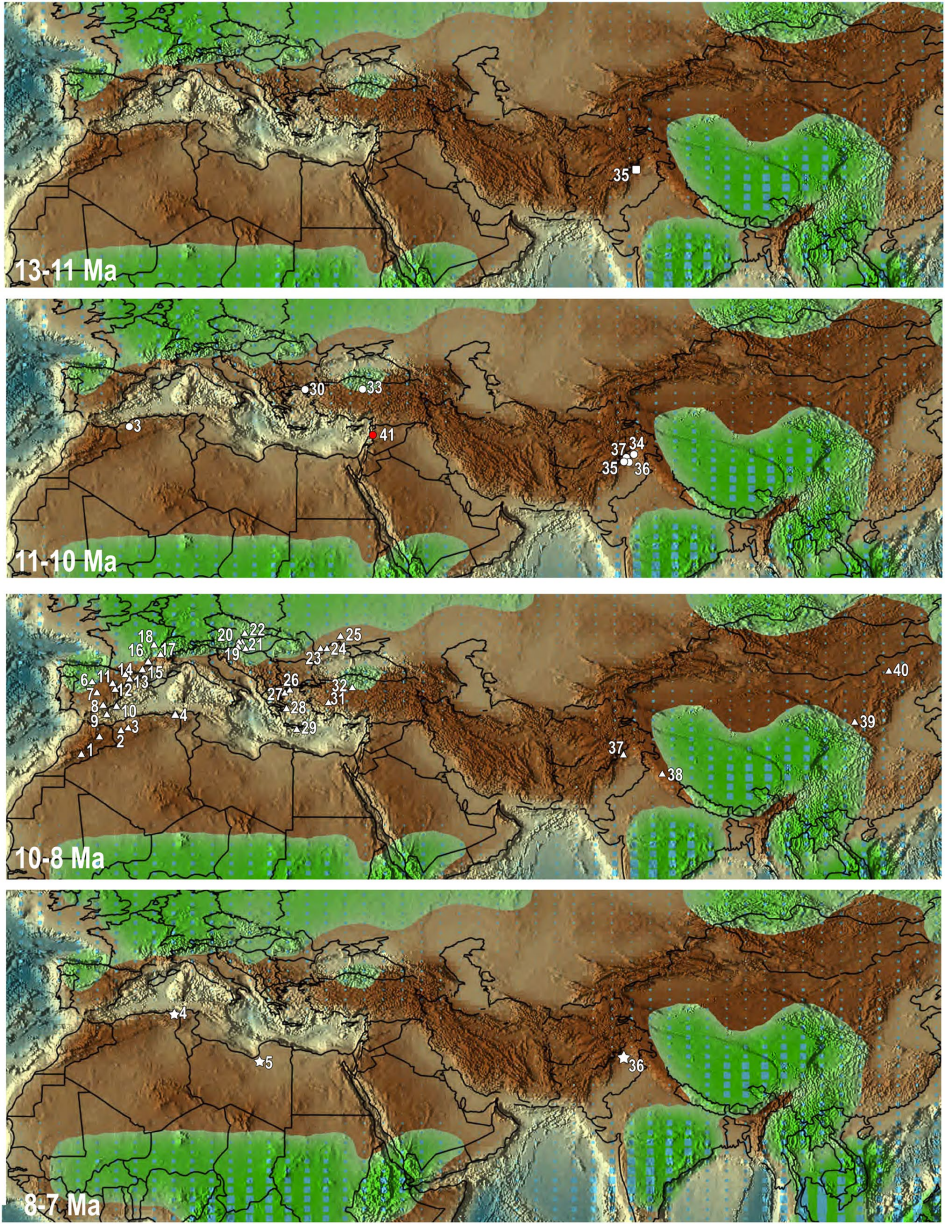
Fossil localities showing the distribution of *Progonomys* in different time intervals plotted in the Late Miocene palaeogeographical map of rainfall of Scottese^54^. **1**, Afoud 6, Oued Tabia, Oued Zra, Wanou (Aït Kandoula Basin, Morocco); **2**, Tafna 2, Feid el Atteuch, (Tafna Basin, Algeria); **3**, Bou Hanifia 5, Sig 2, Sidi Salem (Chelif Basin, Algeria); **4**, El Hiout, Babel Ahmar, Bou Adjeb, El Hiout, Guergour Ferroudj, Maatgua, Ouled el Arbi, Zighout Youcef (Constantine Area, Algeria); **5**, Sahabi (Sirt Basin, Lybia); **6**, Ampudia 3, 9 (Duero Basin, Spain); **7**, Batallones (Madrid Basin, Spain); **8**, Hijar (Hijar Basin, Spain); **9**, Racor (Almería, Spain); **10**, Crevillente (Alicante, Spain); **11**, Cortasogas 2B, Belmonte, Pedregueras 2C (Calatayud-Daroca, Spain); **12**, Cascante Cubla 1, 2, 7/7 A, Peralejos 4, B, C, D, La Gloria 11, Los Aguanaces 5A, 5B, Cañizar 4A, 4B, 6, 9, La Roma 2, Masada Ruea, Puente Minero 1, 2, 8, 10, Masía del Barbo 2A, 2B, Masía de la Roma 4B, 4C, 5, 6, 7, 8, 11 (Teruel Basin, Spain); **13**, Torrent de Febulines, Viladevalls km7, Camí de Can Tarumbot 2, 3, Can Llobateres 2, Autopista de Rubí-Terrassa 7C, 11, Can Cruset, Ronda Oest de Sabadell D6, Can Casablanques, Trinxera de Can Llobateres 0, 1, Trinchera Sur Autopista II, Trinchera Norte Autopista (Vallès Penedès Basin, Spain); **14**, La Bastida (La Seu d’Urgell Basin, Spain); **15**, Castelnou 1B, Lo Fournas 6, 6a, 6b, 6c, 7, 16M (Pyrénées-orientales, France); **16**, Montredon (Midi-Pyrénées, France); **17**, Dionay (Isère, France); **18**, Ambériu 1, 2C, Douvre, Soblay (Ain, France); **19**, Kohfidisch (Pannonian, Austria); **20**, Richardhof-Wald, Neusedl am See, Eichkogel, (Vienna, Austria); **21**, Sümeg (Hungary); **22**, Pezinok A & B (Danube, Slovakia); **23**, Buzhor 1 (Moldova); **24**, Kalfa, Cainari (Moldova); **25**, Mikhalovka 1, 2 (Odessa, Ukraine); **26**, Lefkon (Greece); **27**, Ravin de la Pluie (Greece); **28**, Biodrak (Greece); **29**, Kastellios K1, K3 (Krete); **30**, Bayraktepe 1, 2 (Turkey); **31**, Altintas 1, 2, Kütahya A, B (Kütahya, Turkey); **32**, Localities 8A, 84 (Sinap Tepe, Turkey); **33**, Tuglu 6/7, Güney, Mahmutkoy (Çankiri, Turkey); **34**, Jalapur 101 (Jalapur, Pakistan); **35**, localities 76, 83, 311, 504, 534, 797, 809 (Sethi Nagri, Pakistan); **36**, localities 450, 921 (Hasnot, Pakistan); **37**, localities 24, 34, 182, 259, 367, 388 (Khaur, Pakistan); **38**, Ladhyani (Bilaspur, India); **39**, Lantian 12, 13, 18, 19, 20, 38 (Lantian, China); **40**, Juhr (Shala, China); **41**, Zahleh (Bekaa Valley, central Lebanon), where *Progonomys manolo* has been discovered, in red. Light blue squares = precipitation; green = wet areas where precipitation > evaporation; tan = drier areas.

The fossil record is crucial in providing unequivocal data on past distributions and dispersal events of extinct organisms. Fossil remains of *Progonomys* provide evidence of large scale dispersals between and within Eurasia and Africa (Fig. 7). They suggest that the origin of *Progonomys* is in Pakistan (Potwar Plateau, Fig. 7a). A predecessor of the genus has been indeed found in fossiliferous localities of this area dated 12.4–12.3 Ma^4^. The dispersals of *Progonomys* have been considered to be diachronic, particularly in Anatolia, where the earliest record of *Progonomys* was supposed to be that of Sinap with an estimated age of 10.1 Ma^7^. However, new palaeontological data together with stratigraphic reinterpretations of Late Miocene deposits in the Çankırı-Çorum Basin reveal that *Progonomys* is already present near the base of the Tuḡlu Formation (11–10.5 Ma)^22^. Therefore, the arrival of this taxon in Anatolia took place earlier (Fig. 7b). These data are in accordance with findings of *Progonomys* in eastern Europe, in Moldova at Buzhor and Kalfa (11–9.78 Ma) and Cainari (~10 Ma), and in Ukraine at Mikhalovka (*circa* 9.78 Ma)^23,24^ (Fig. 7b). It is noteworthy that no *Progonomys* remains have been found in Russia (the Muridae gen. indet. from Maikop (Russia)^25^, which was referred to as cf. *Progonomys*^24^, belongs, in fact, to *Parapodemus*^26)^. The oldest remains of *Progonomys* in south-eastern Europe (Greece) are younger (9.6 Ma)^27,28^ (Fig. 7c) than those from eastern Europe, but no localities correlated with MN9 are known in Greece^28^. Therefore, the arrival of *Progonomys* in Greece may have taken place earlier than 9.6 Ma. The spread into central and western Europe seems to have been delayed with respect to eastern Europe. In Slovakia, Hungary and Austria, remains of *Progonomys* of about 9.7 Ma have been found^18,29–31^, whereas the record of this genus is somewhat older in France and Spain (9.97 Ma)^32,33^ (Fig. 7c).

The dispersal of *Progonomys* from Pakistan towards East Asia, where the earliest record dates 10 Ma^34^, seems to have occurred later (Fig. 7c).

*Progonomys* entered central and western Europe about one million years later than northern Africa, where the oldest remains date 10.8 Ma: Algeria^13,35^ and, possibly, Egypt^36,37^. The genus may have spread subsequently into Morocco, where it has been reported from some localities^12,13,38^ dated *circa* 9.7 Ma^35^. Its most recent record (*Progonomys* sp. according to Munthe^39^, *Progonomys* aff. *mauretanicus* according to Agustí^40^) on the African continent is from the Libyan site of As Sahabi^39^. As Sahabi is considered to date 7.3–7.5 Ma^41^ but without certainty (see also Agusti^40^ and El-Shawaihdi *et al*.^42^). The Libyan *Progonomys* together with the most recent ones from the Siwaliks^3^ may represent the last known occurrence of *Progonomys* globally.

The main biogeographical pattern of *Progonomys* consists in long distance dispersal events from southern Asia to eastern Asia and western Asia, from there to eastern Europe (10.5–11 Ma) and farther across central and western Europe (*circa* 10 Ma) on one side, and through the Levantine corridor to reach northern Africa on the other. The spread of *Progonomys* out of Pakistan westwards might have been triggered by changes in the eustatic sea level. The sea level gradually fell during the Middle Miocene, until the Middle-Late Miocene boundary^43,44^ (11.6 Ma^45^), when it drastically dropped. This Serravallian-Tortonian sea level fall has been documented worldwide^44^ and associated with the establishment of the East Antartic Ice Sheet^46^. The arrival of *Progonomys* in the Arabian Peninsula seems to be coeval with the final closure of the eastern Tethys and the end of marine connection between the Mediterranean Sea and the Indian Ocean along northern Arabia, which has been estimated to occur at *circa* 11 Ma^47^.

The existence of marine barriers between northern Morocco and southern Spain, which connected the Mediterranean Sea with the Atlantic Ocean during the Tortonian^48,49^, would have prevented the dispersal of *Progonomys* in western Europe from the Maghreb and *vice versa*. The new species of *Progonomys* from Lebanon that we have described herein constitutes the first record of this genus in the whole Arabian Peninsula and circumambient areas and the first physical evidence that the dispersal of *Progonomys* from Asia to Africa took place through the Levant.

About one million years after its dispersal from Pakistan to the west, *Progonomys* expanded towards eastern Asia (*circa* 10 Ma). *Progonomys* would have remained in an area of open environments, which it seems to have favoured as suggested by the global distribution of this taxon displayed in the Late Miocene palaeogeographical map of rainfall^50^ (Fig. 7). The fact that the distribution of *Progonomys* was restricted to the southern half of the Paleartic Region speaks volumes about the ecological requirements of this taxon. The Late Miocene Palaeartic realm was characterized by seasonal and open environments, whereas the Oriental realm had a subtropical and moister climate^51^. It is plausible that the shift from moist conditions to drier and more open environments in the Pakistan area that took place at the Middle-Late Miocene boundary^52–54^ triggered the appearance of the first *Progonomys*-like morphologies (‘near *Progonomys*’^4^) from *Antemus* or an allied form.

Morphometric studies on the first molars of extinct and extant murines^55–62^ have allowed exploring the correlations between outline shape, climate and diet. The morphometric analyses carried out in the present work provide evidence of differences in size and shape through time within the genus *Progonomys*. Primitive morphologies that correspond to the geologically oldest representatives of the genus (*Progonomys hussaini*, *Progonomys ibrahimi*, *Progonomys morganae*, early *Progonomys hispanicus*, *Progonomys manolo*) are characterized by a very elongate outline of M1. In contrast, latest *Progonomys* (*Progonomys cathalai*, *Progonomys clauzoni*, late *Progonomys hispanicus* or *Progonomys minus*) are characterized by a broader outline of M1. In murines, slender and asymmetrical shapes have usually been related to an omnivorous and generalist diet, whereas broader and more symmetrical outlines have been related to more exclusively herbivorous feeding habits^55–57,61^. Even if the changes in the outline of the molars of *Progonomys* were not related to the development of crests, those taxa having broader molars (and, therefore, larger occlusal surfaces for the same jaw length) would have been better adapted to a more fibrous diet. This agrees with the results of microwear analyses^60^, which suggest a diet close to that of grass-dominated feeders for *Progonomys castilloae*, *Progonomys cathalai* and late *Progonomys hispanicus*. In fact, tooth shape may have changed through time in *Progonomys* as a response to an increase of aridity and more open environments about 10.5 Ma on a global scale^63–72^. So, the oldest representatives of the genus were most probably generalist omnivores with a plant-dominated diet and subsequently acquired a more specialized herbivorous diet before going extinct 7.4 Ma, when a period of greater aridity occurred (evidenced by the decline of trees and shrubs, C3 vegetation, and the dominance of warm season grasses, C4 vegetation^46^). Thus, both the appearance and extinction of *Progonomys* may have been triggered by episodes of climatic shifts that resulted in increasingly open environments.

## Conclusion

*Progonomys manolo* is a primitive murine with small cheek teeth that lack any remnant of longitudinal connections between their cusps. The outline of the first upper molars in occlusal view is slender and fairly asymmetrical. The upper molars lack cusp t7 and have the posterior cingulum well developed; the lower molars show well-developed cingulum cuspids. The discovery of this new taxon has allowed narrowing down the estimated age of the “Zahleh Formation”: its deposition may have started as early as the early Tortonian (10.5–11 Ma). *Progonomys* was the first true murine that dispersed out of Asia. By 11 Ma, this taxon extended its range from southern Asia through western Asia to reach Eastern Europe, from where it spread across central and western Europe (about 10 Ma). The first record of a species of *Progonomys* in the Levant enhances the importance of the “Levantine Corridor” as a crossroad between Eurasia and Africa and sheds light on the oldest known intercontinental dispersal of Murinae. *Progonomys manolo* is part of a successful dispersal phenomenon that gave rise to the populations of *Progonomys* that later lived in Africa. The spread of *Progonomys* out of Pakistan was probably allowed by the dramatic lowering of sea level that took place at the Middle-Late Miocene boundary (~11.6 Ma). The restriction of this genus to the southern half of the Palaearctic Zoogeographical Realm reflects its ecological requirements. In this part of the world, a climatic transition from warm and moist (Middle Miocene) to increasingly drier (Late Miocene) seems to have had an important effect in the evolution of *Progonomys* since its appearance (~12 Ma) till its extinction (~7.4 Ma). The slender and fairly asymmetrical outline of the first upper molar of *Progonomys manolo* still points to a generalist and omnivorous diet for this species. Later evolution of *Progonomys* toward molars with broader outline indicates a change to more specialised feeding preferences (including abrasive and fibrous plants) that will only strengthen with the development of the stephanodont dental pattern in succeeding new murine genera (e.g., *Occitanomys* and *Stephanomys*).

## Methods

### Specimens

We examined teeth of extant Phloeomyini (*Batomys granti*, *Phloemoys cumingi*), Malacomini (*Malacomys longipes, Malacomys* sp.), Murini (*Mus musculus*, *Mus minutoides*, *Mus spretus*), Hydromyini (*Anisomys imitator, Conilurus penicillatus, Leggadina forresti, Leporillus conditor, Mastacomys fuscus, Mesembriomys gouldi, Notomys alexis, Notomys longicaudatus, Pseudomys australis, Rhinchomys soricoides, Xeromys myoides, Zizomys argurus*), Rattini (*Dacnomys millardi, Berylymys bowersi mackenziei, Leopoldamys sabanus, Maxomys bartelsii, Maxomys surifer, Maxomys whiteheadi, Nivivinter cremoriventer, Nivivinter culturatus, Sundamys muelleri, Rattus villosissimus, Rattus exularis, Rattus rattus, Rattus exulans, Rattus norvegicus*), Apodemyini (*Apodemus sylvaticus, Apodemus flavicollis, Apodemus agrarius*, *Apodemus mystacinus, Apodemus semotus*), Praomyini (*Hylomiscus parvus, Mastomys natalensis, Praomys jacksoni, Praomys tulbergi, Zelotomys hildergardeae*) housed in the zoological collections of the Natural History Museum, London (NHM), and the Museo Nacional de Ciencias Naturales-CSIC, Madrid (MNCN-CSIC), as well as fossil teeth of *Progonomys hispanicus*, *Progonomys castilloae, Progonomys clauzoni, Progonomys cathalai* and *Progonomys woelferi* housed in the palaeontological collections of the University Claude Bernard, Lyon, and the University of Montpellier, Montpellier, as well as casts of specimens of *Progonomys hussaini*, *Progonomys debruijni* and *Mus auctor* housed in the palaeontological collections of the University of Montpellier.

This work is based on isolated molars of early murines obtained by screen-washing (with a mesh of 0.5 mm) and sorting of about 2 tons of sediment. The new specimens have been described and compared with most relevant representatives of Murinae. However, a detailed comparison has only been carried out with the species considered as belonging to the genus *Progonomys*, to which the Lebanese taxon clearly belongs. First, second and third lower molars are designated as m1, m2 and m3, respectively, and first, second and third upper molars as M1, M2 and M3, respectively. The terminology used in the tooth descriptions follows that of Jacobs^73^ (Fig. 1), which is not only consistent in its main aspects with that commonly used for other mammals, but also reflects the major homology of cusps between murines and cricetines^73^. The occlusal measurements (greatest length and greatest width; Supplementary Tables S1 and S2) of the teeth of the taxon from Lebanon have been obtained with a Nikon digital counter CM-6S measuring device.

### Morphometrics

Nine first upper molars (M1) from Zahleh were considered for morphometric analyses. They were compared to a set of rodent molars of Miocene age documenting the following genera: *Antemus*, *Castillomys*, *Huerzelerimys*, *Karnimata*, *Mus*, *Occitanomys*, *Parapodemus*, *Parapelomys*, *Progonomys* (including *Sinapodemus*) (Supplementary Table S3). The data set includes a total of 541 M1. Data resulting from the analysis of the cheek teeth of *Huerzelerimys*, *Occitanomys*, European *Parapodemus* and *Progonomys* correspond to previously published morphometric analyses^55,59^ (see Supplementary Table S3). *Castillomys crusafonti* has been documented on the basis of M1 from Sète (Pierre Mein collection), France, housed in the paleontological collections of the University Claude Bernard, France^74^. Data obtained for the first upper molars of the Siwalik murines (*Antemus chinjiensis, Karnimata darwini, Karnimata huxleyi, Mus auctor, Parapelomys robertsi* and *Progonomys debruijni*) come from photos kindly provided by Yuri Kimura and those from *Progonomys hussaini, Progonomys morganae, Parapodemus badgleyae* and *Karnimata fejfari* are from Kimura *et al*.^4^. Data from the first upper molars of *Progonomys sinensis, Progonomys shalaensis*, *Progonomys ibrahimi* and *Progonomys minus* result from measurements taken from the plates of Qiu *et al*.^75^, Qiu and Li^76^ and Sen^15^, respectively.

First upper molars have been photographed so that the occlusal surface matches best the horizontal plane. Based on these photographs, the shape of the M1 was described using 64 points sampled at equal curvilinear distance along their 2D outline. The starting point was tentatively positioned towards the anteriormost part of the tooth. Using the Momocs package^77^, the outlines were then aligned along their long axis, and the starting point slid towards the uttermost point along this long axis, consistently adjusting its position at the most anterior part of the tooth.

From the 64 points, 64 radii (i.e. distance of each point to the centre of gravity of the outline) were computed. Expressed as a function of the curvilinear distance along the outline, this set of radii constituted a function that was analysed using a Fourier analysis. Accordingly, the empirical function is decomposed into a sum of trigonometric functions of decreasing wavelength (the harmonics). Each is weighted by two Fourier coefficients (FCs), which constitute the shape variables to be compared among individuals. The zero harmonic (A0) is proportional to the size of the outline. It was used to standardize all other FCs so that they represent shape only. This Fourier analysis was performed using the newly implemented ‘sfourier’ function in Momocs. The higher the rank of the harmonics, the more details they represent on the outline^78,79^ and the less information they bring. This can be used to filter measurement error and reduce the number of variables, by discarding high-order harmonics^80^. Considering the cumulative power (i.e. information brought by each successive harmonics), it appeared that the molar tooth could be adequately described by the first seven harmonics, i.e. by 14 variables (FCs), representing more than 99% of the total information.

The 64 points along the outline are enclosed into a bounding box (i.e. the smallest rectangle that enclosed the object delineated by the points). The length and width of this bounding box provided an estimate of these dimensions for each tooth (function coo_lw in Momocs).

The shape of each molar was described by a multivariate dataset (14 FCs). A Principal Component Analyses (PCA) on the variance-covariance matrix of the FCs was performed to represent the total variance on few synthetic shape axes. Relationships between groups (species per deposits) were further assessed by calculating the Euclidean distances between the group means of the 14 FCs. An unrooted neighbor-joining tree allowed a representation of these relationships (Fig. 6).

Differences in univariate parameters (length, width and length/width ratio) between the Zahleh sample and the genera identified as candidates as its closest relatives were further tested using t-tests, which perform well even with very small sample sizes^81^.

The morphological variation in the Zahleh sample (excluding Zahleh 02) was assessed as the coefficient of variation (= standard deviation/mean) for the M1 length, the variance of the ratio length/width and the shape variance (= sum of the variance of the 14 Fourier coefficients). Ten bootstrap estimates assessed the uncertainty related to sampling. These estimates of within-sample variation were compared to those observed for *Mus auctor* (n = 7) and for a series of *Progonomys* samples: *Progonomys cathalai* from Soblay (n = 38), *Progonomys hispanicus* from La Roma 4c (n = 8) and Masia del Barbo (n = 20), *Progonomys clauzoni* from Lo Fournas 6 (n = 67) and *Progonomys castilloae* from Lo Fournas 7 (n = 46). Various modern populations from the wood mouse *Apodemus sylvaticus* allowed the estimation of the variation in a context in which interspecific mixing can be definitely discarded based on genetic analyses. The sample from Mimizan (AS-Mim, n = 63) included specimens from four neighbouring populations and the sample from Lantabat (AS-Lant, n = 41) included seasonal variation within a population. A third sample (AS-geo, n = 15 + 15 + 15) included specimens from Belgium, France and Spain to cover geographic variation^82^.

For all these samples, the size and shape variation was estimated on the original dataset and on 10 bootstrapped datasets. In samples with n > 10, the sample size was rarefied to n = 10, in order to be comparable to the limited sample size in Zahleh (n = 8).

Multivariate analyses, statistical analyses and representations were performed using Momocs, ade4^83^ and ape^84^ under R^85^.

### Nomenclatural acts

This published work and the nomenclatural acts it contains have been registered in ZooBank, the proposed online registration system for the International Code of Zoological Nomenclature (ICZN). The ZooBank LSIDs (Life Science Identifiers) can be resolved and the associated information viewed through any standard web browser by appending the LSID to the prefix ‘http://zoobank.org/’. The LSIDs for this publication are urn:lsid:zoobank.org:pub:XXXX, urn:lsid:zoobank.org:act:YYYYY and urn:lsid:-zoobank.org:act:ZZZZ.

## Supplementary information

Supplementary Information
