# Supplementary material for: First levantine fossil murines shed new light on the earliest intercontinental dispersal of mice
Source: Sci Rep. 2019 Aug 29;9:11874. doi: 10.1038/s41598-019-47894-y (PMC6715647; doi:10.1038/s41598-019-47894-y)
Supplement: Supplementary file 1 — Supplementary Information [file 41598_2019_47894_MOESM1_ESM.pdf]

SUPPORTING INFORMATION

First levantine fossil murines shed new light on the earliest intercontinental dispersal of mice

Raquel López-Antoñanzas[1,2]*, Sabrina Renaud[3], Pablo Peláez-Campomanes[2], Dany Azar[4,] George Kachacha[4] & Fabien Knoll[5,6]

[1]Laboratoire de Paléontologie, Institut des Sciences de l'Évolution (UMR-CNRS 5554), Montpellier, France

[2]Departamento de Paleobiología, Museo Nacional de Ciencias Naturales-CSIC, Madrid, Spain

[3]Laboratoire de Biométrie et Biologie Evolutive, UMR 5558, CNRS, Université Lyon 1, 69622 Villeurbanne, France

[4]Natural Sciences Department, Faculty of Sciences II, Lebanese University, Fanar, Lebanon

[5]ARAID—Fundación Conjunto Paleontológico de Teruel-Dinopolis, 44002 Teruel, Spain

[6]School of Earth and Environmental Sciences, University of Manchester, Manchester, United Kingdom

Supplementary Figure S1 | Interactive visualization made from the surface rendering of CT scan of the holotype and several paratypes of *Progonomys manolo* sp. nov.

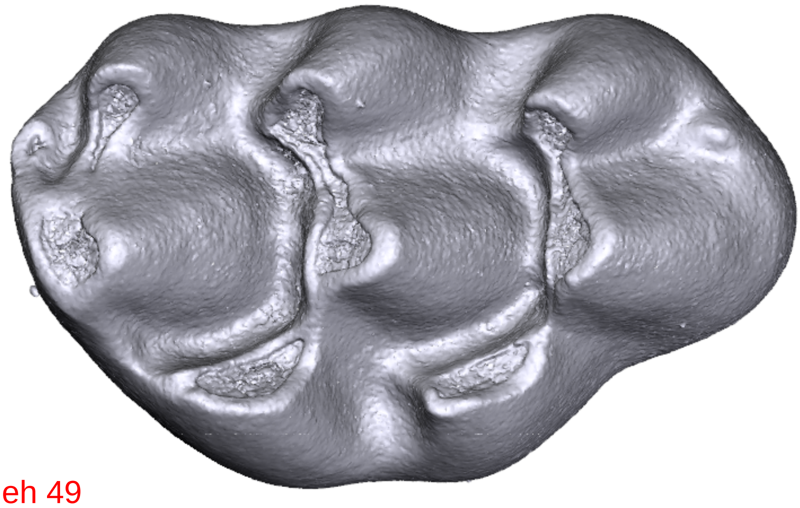

Zahleh 49

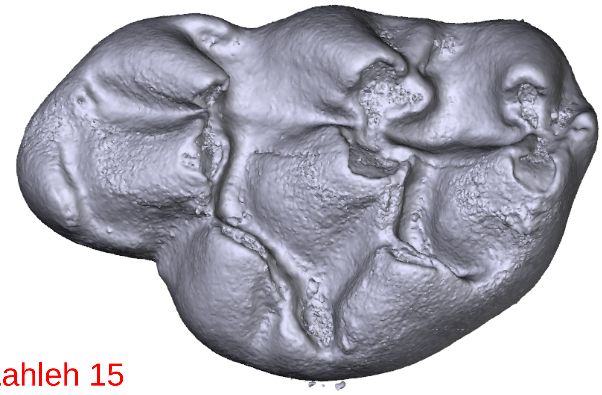

Zahleh 15

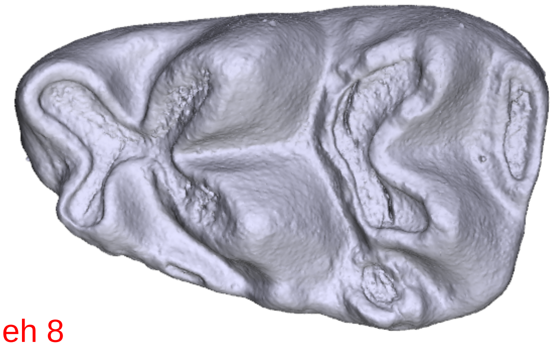

Zahleh 8

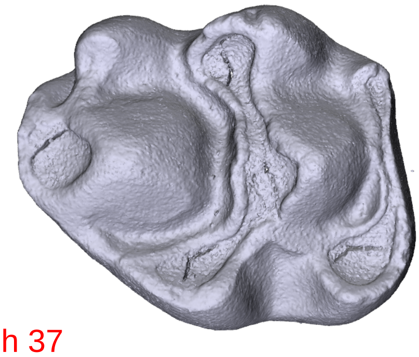

Zahleh 37

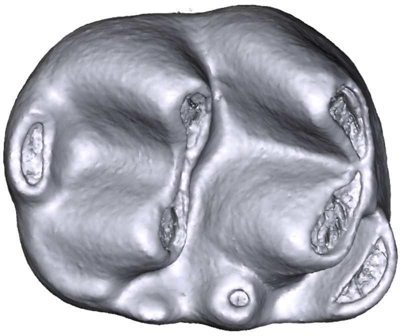

Zahleh 44

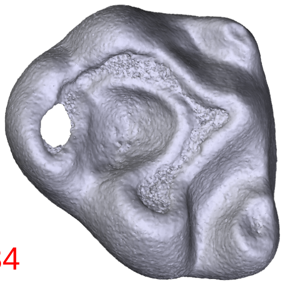

Zahleh 34

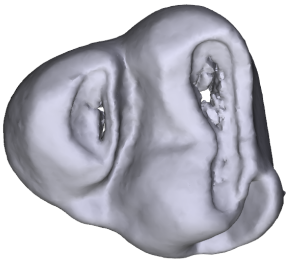

Zahleh 86

Supplementary Figure S2 | SEM of the occlusal surface of the M1 of the murine specimen Zahleh 02. Scale bar equals 500 µ.

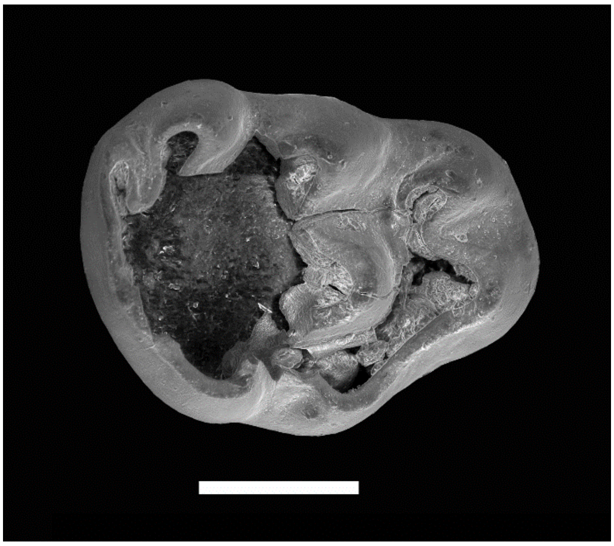

Supplementary Figure S3 | Original and bootstrapped estimates of the morphological variation among the specimens from Zahleh and a series of modern wood mouse populations and fossil samples.

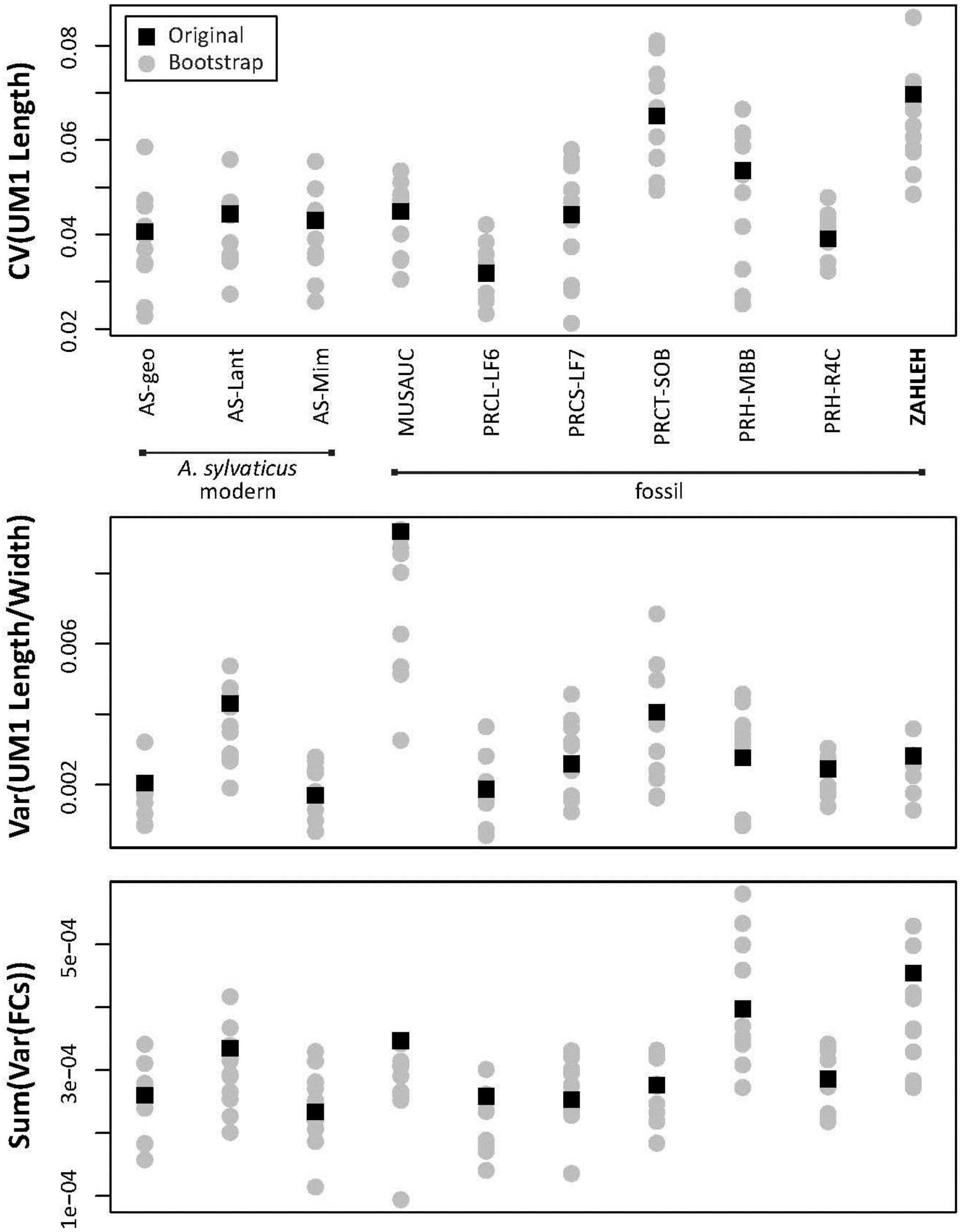

Supplementary Figure S4 | Scatter diagrams (length and width) of the lower and upper molars of *Progonomys* spp. in comparison with the new Lebanese taxon *Progonomys manolo*.

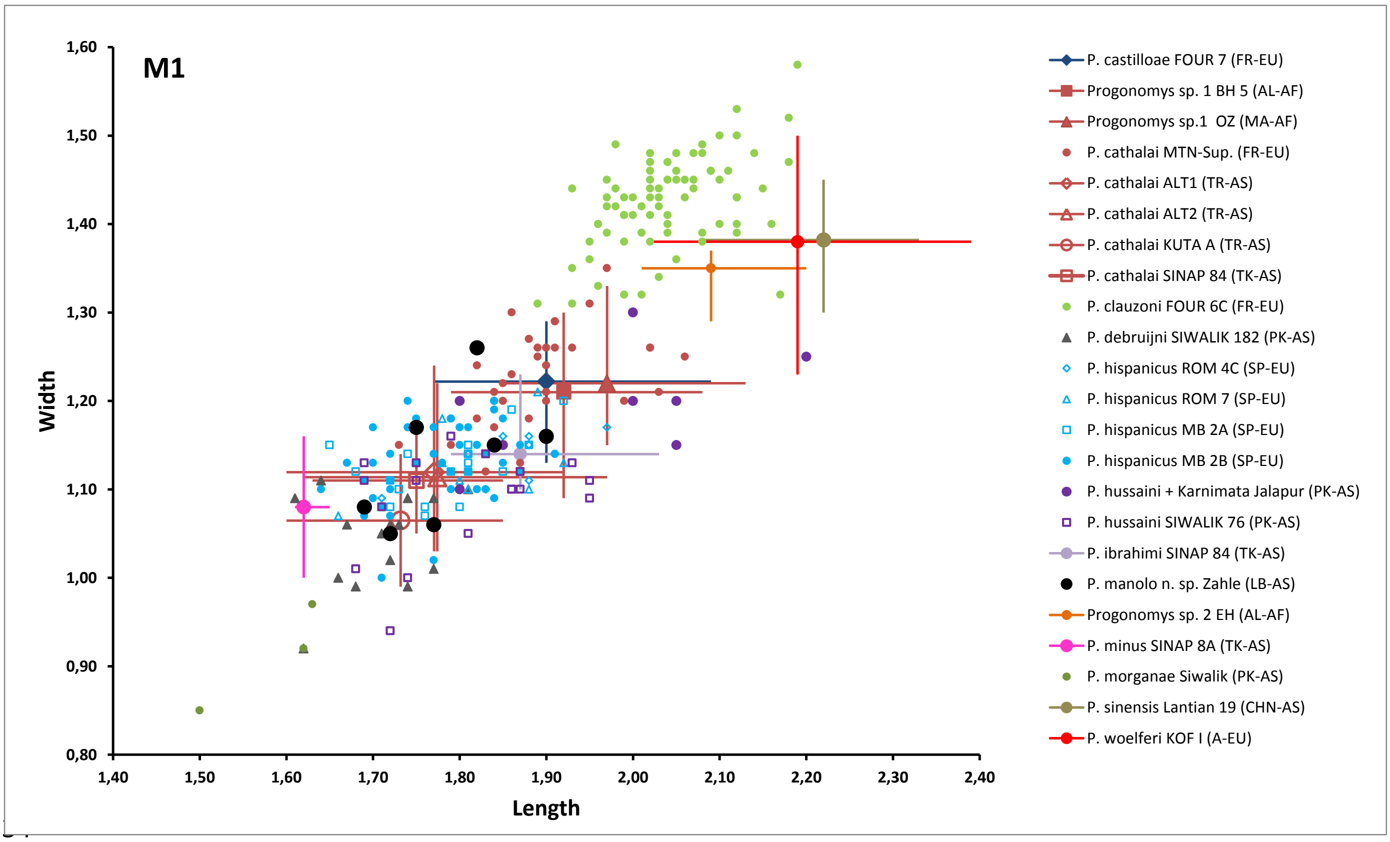

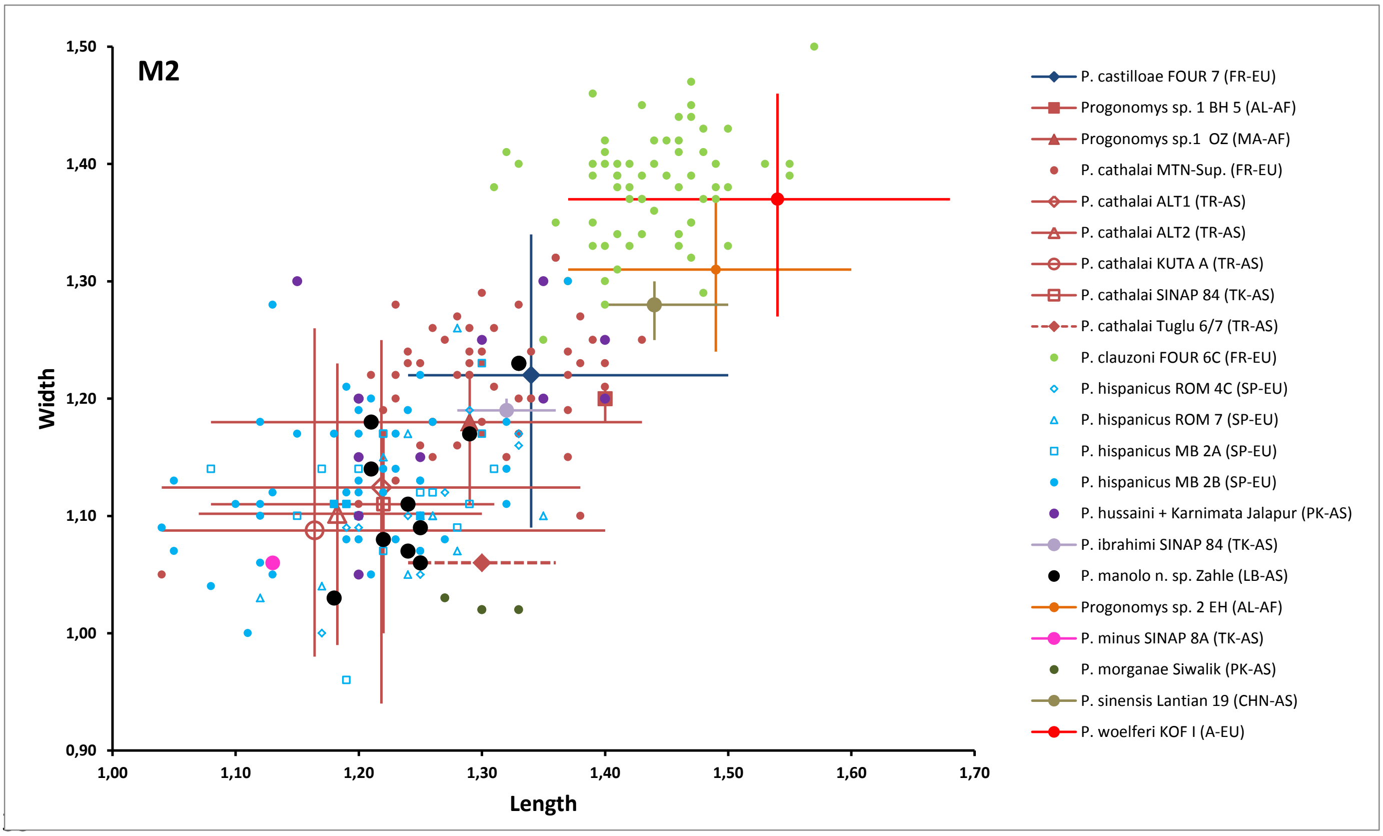
M2
Width
Length
1,50
1,40
1,30
1,20
1,10
1,00
0,90
1,00
1,10
1,20
1,30
1,40
1,50
1,60
1,70
P. castilloae FOUR 7 (FR-EU)
Progonomys sp. 1 BH 5 (AL-AF)
Progonomys sp.1 OZ (MA-AF)
P. cathalai MTN-Sup. (FR-EU)
P. cathalai ALT1 (TR-AS)
P. cathalai ALT2 (TR-AS)
P. cathalai KUTA A (TR-AS)
P. cathalai SINAP 84 (TK-AS)
P. cathalai Tuglu 6/7 (TR-AS)
P. clauzoni FOUR 6C (FR-EU)
P. hispanicus ROM 4C (SP-EU)
P. hispanicus ROM 7 (SP-EU)
P. hispanicus MB 2A (SP-EU)
P. hispanicus MB 2B (SP-EU)
P. hussaini + Karnimata Jalapur (PK-AS)
P. ibrahimi SINAP 84 (TK-AS)
P. manolo n. sp. Zahle (LB-AS)
Progonomys sp. 2 EH (AL-AF)
P. minus SINAP 8A (TK-AS)
P. morganae Siwalik (PK-AS)
P. sinensis Lantian 19 (CHN-AS)
P. woelferi KOF I (A-EU)

36

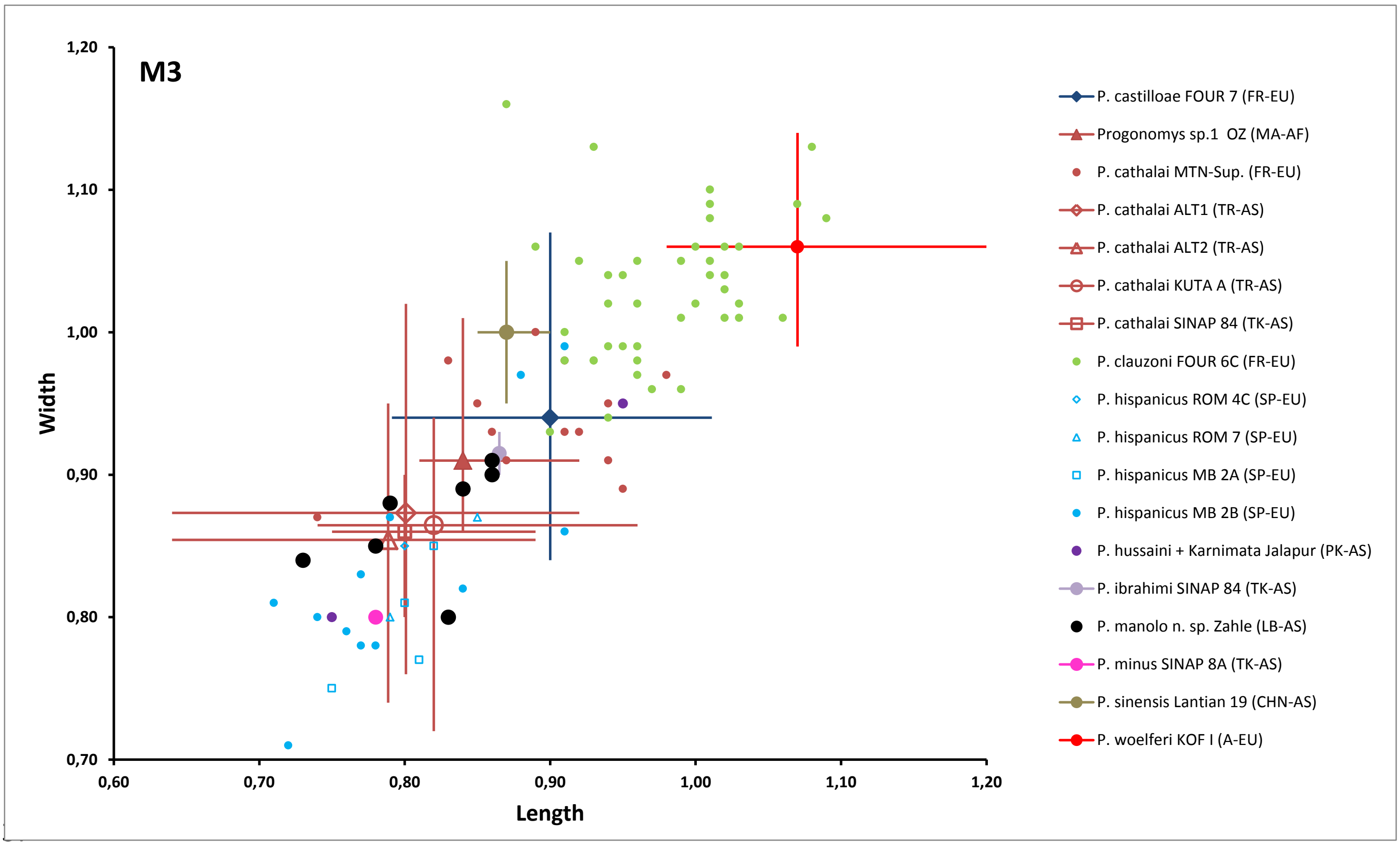
M3
Width
Length
1,20
1,10
1,00
0,90
0,80
0,70
0,60
0,70
0,80
0,90
1,00
1,10
1,20
P. castilloae FOUR 7 (FR-EU)
Progonomys sp.1 OZ (MA-AF)
P. cathalai MTN-Sup. (FR-EU)
P. cathalai ALT1 (TR-AS)
P. cathalai ALT2 (TR-AS)
P. cathalai KUTA A (TR-AS)
P. cathalai SINAP 84 (TK-AS)
P. clauzoni FOUR 6C (FR-EU)
P. hispanicus ROM 4C (SP-EU)
P. hispanicus ROM 7 (SP-EU)
P. hispanicus MB 2A (SP-EU)
P. hispanicus MB 2B (SP-EU)
P. hussaini + Karnimata Jalapur (PK-AS)
P. ibrahimi SINAP 84 (TK-AS)
P. manolo n. sp. Zahle (LB-AS)
P. minus SINAP 8A (TK-AS)
P. sinensis Lantian 19 (CHN-AS)
P. woelferi KOF I (A-EU)

38

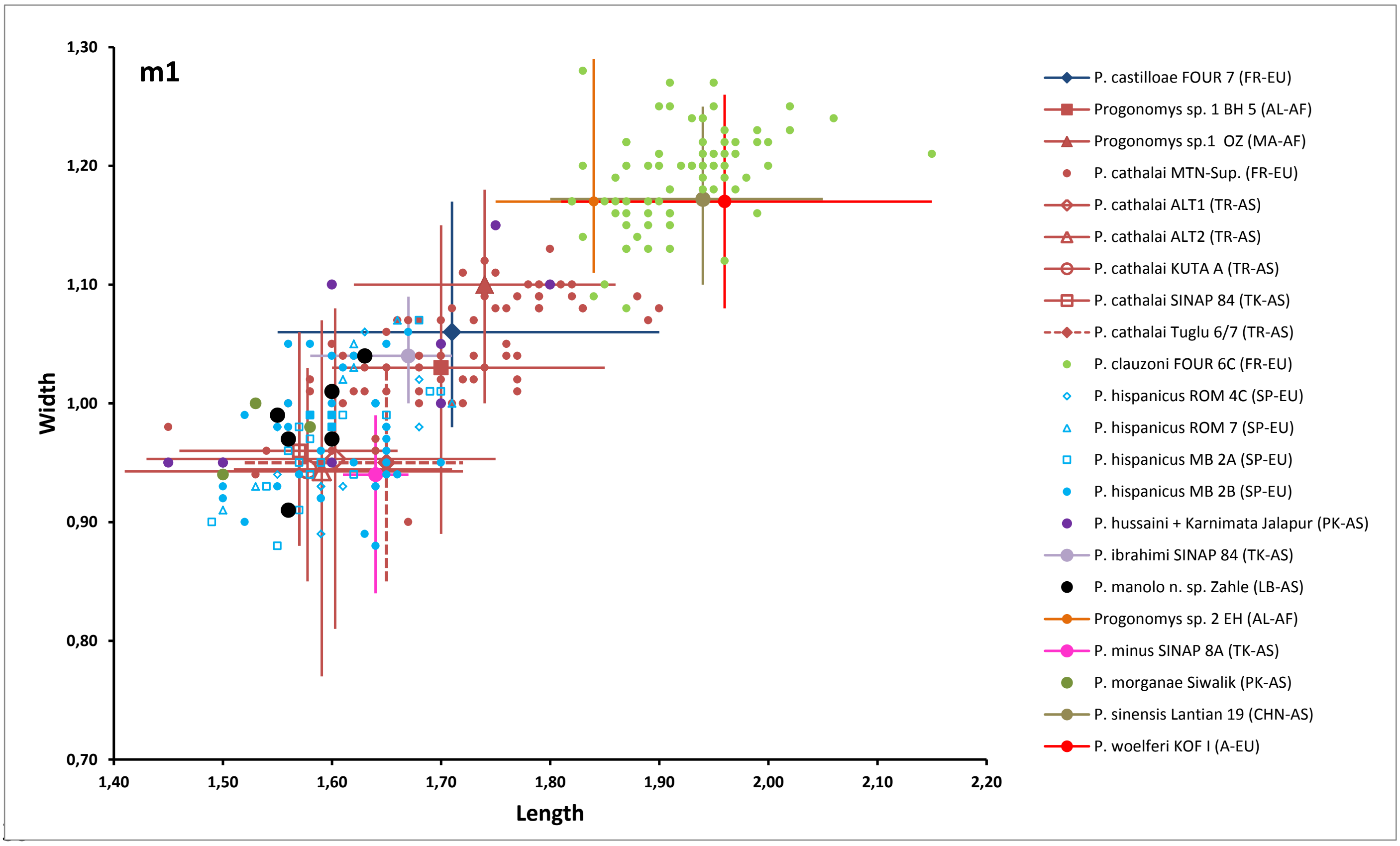

m1
Width
Length
1,30
1,20
1,10
1,00
0,90
0,80
0,70
1,40
1,50
1,60
1,70
1,80
1,90
2,00
2,10
2,20
P. castilloae FOUR 7 (FR-EU)
Progonomys sp. 1 BH 5 (AL-AF)
Progonomys sp.1 OZ (MA-AF)
P. cathalai MTN-Sup. (FR-EU)
P. cathalai ALT1 (TR-AS)
P. cathalai ALT2 (TR-AS)
P. cathalai KUTA A (TR-AS)
P. cathalai SINAP 84 (TK-AS)
P. cathalai Tuglu 6/7 (TR-AS)
P. clauzoni FOUR 6C (FR-EU)
P. hispanicus ROM 4C (SP-EU)
P. hispanicus ROM 7 (SP-EU)
P. hispanicus MB 2A (SP-EU)
P. hispanicus MB 2B (SP-EU)
P. hussaini + Karnimata Jalapur (PK-AS)
P. ibrahimi SINAP 84 (TK-AS)
P. manolo n. sp. Zahle (LB-AS)
Progonomys sp. 2 EH (AL-AF)
P. minus SINAP 8A (TK-AS)
P. morganae Siwalik (PK-AS)
P. sinensis Lantian 19 (CHN-AS)
P. woelferi KOF I (A-EU)

40

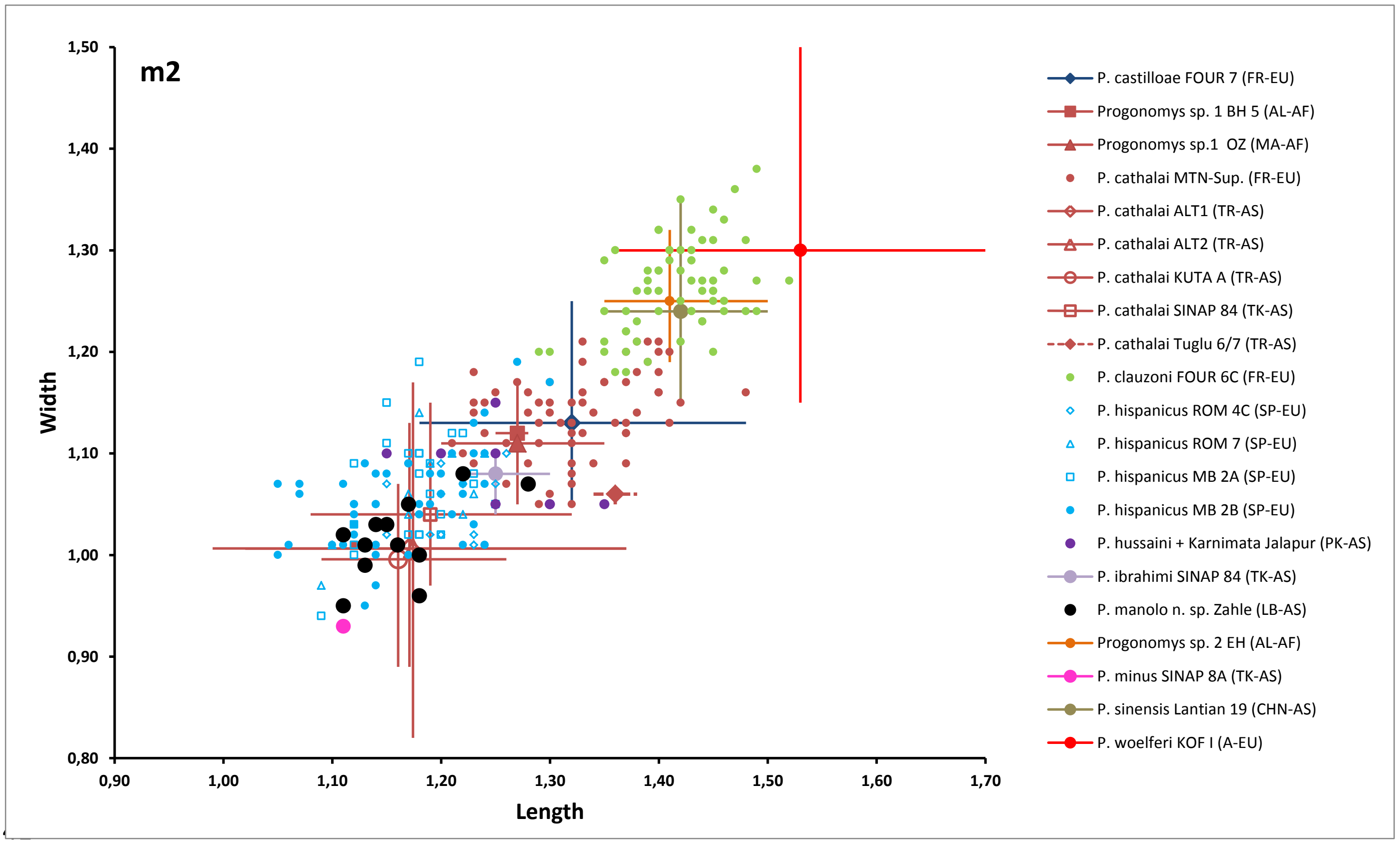

m2
Width
Length
1,50
1,40
1,30
1,20
1,10
1,00
0,90
0,80
0,90
1,00
1,10
1,20
1,30
1,40
1,50
1,60
1,70
P. castilloae FOUR 7 (FR-EU)
Progonomys sp. 1 BH 5 (AL-AF)
Progonomys sp.1 OZ (MA-AF)
P. cathalai MTN-Sup. (FR-EU)
P. cathalai ALT1 (TR-AS)
P. cathalai ALT2 (TR-AS)
P. cathalai KUTA A (TR-AS)
P. cathalai SINAP 84 (TK-AS)
P. cathalai Tuglu 6/7 (TR-AS)
P. clauzoni FOUR 6C (FR-EU)
P. hispanicus ROM 4C (SP-EU)
P. hispanicus ROM 7 (SP-EU)
P. hispanicus MB 2A (SP-EU)
P. hispanicus MB 2B (SP-EU)
P. hussaini + Karnimata Jalapur (PK-AS)
P. ibrahimi SINAP 84 (TK-AS)
P. manolo n. sp. Zahle (LB-AS)
Progonomys sp. 2 EH (AL-AF)
P. minus SINAP 8A (TK-AS)
P. sinensis Lantian 19 (CHN-AS)
P. woelferi KOF I (A-EU)

42

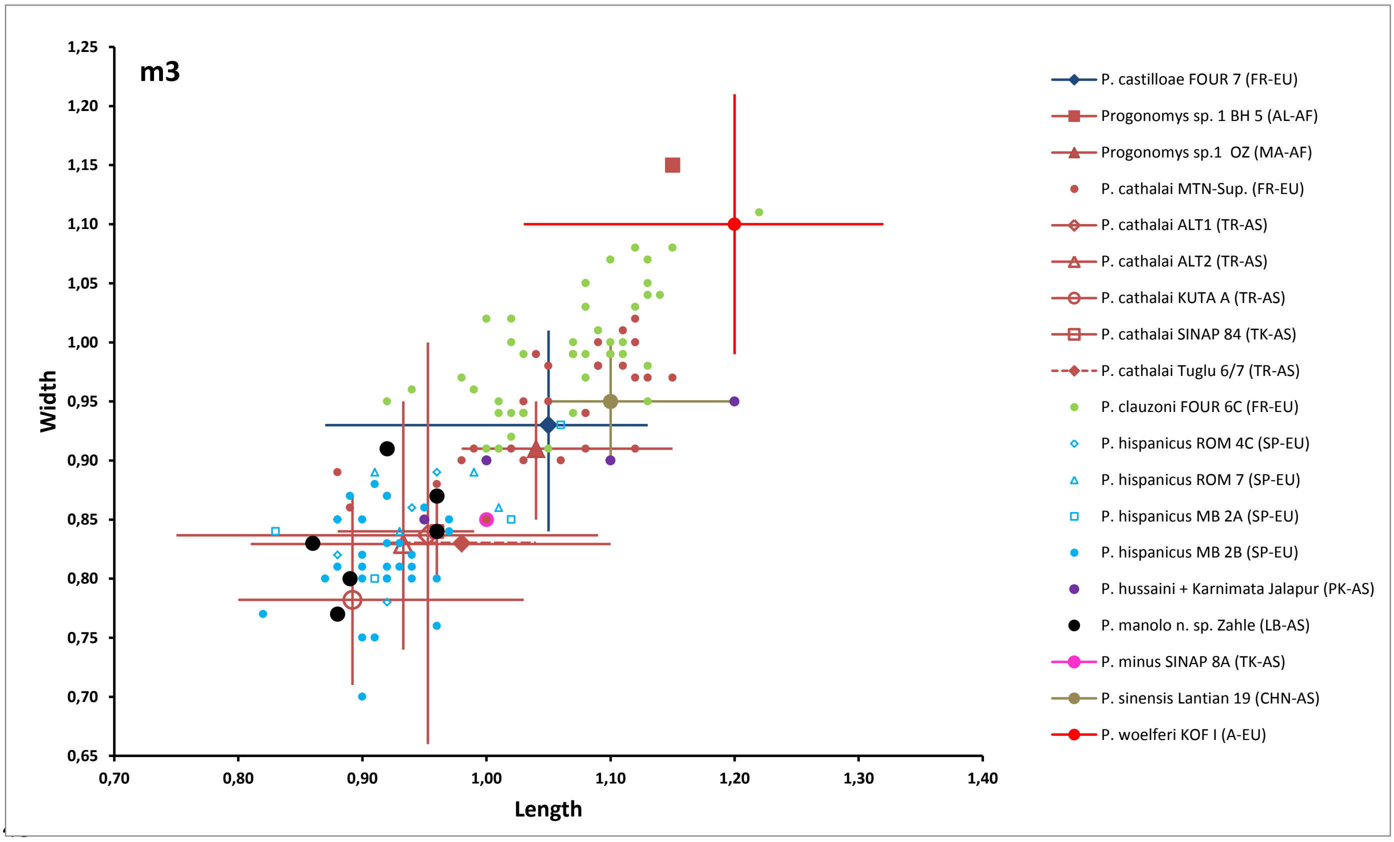

Supplementary Table S1 | Occlusal measurements (mm) of the teeth of *Progonomys manolo* sp. nov. All measurements represent greatest length and greatest width.

Element	Locality	Cat. number	Length	Width
M1	Zahleh	5	1.82	1.26
M1	Zahleh	19	1.77	1.06
M1	Zahleh	31	/	1.14
M1	Zahleh	77	/	1.17
M1	Zahleh	78	1.72	1.05
M1	Zahleh	80	/	/
M1	Zahleh	83	/	/
M1	Zahleh	90	/	/

M1	Zahleh	94	1.75	1.17
M1	Zahleh	95	1.84	1.15
M1	Zahleh	134	1.9	1.16
M1	Zahleh	165	1.69	1.08
M2	Zahleh	37	1.18	1.03
M2	Zahleh	48	1.21	1.18
M2	Zahleh	52	/	/
M2	Zahleh	76	1.33	1.23
M2	Zahleh	82	1.22	1.08
M2	Zahleh	88	1.25	1.09
M2	Zahleh	92	1.33	1.23
M2	Zahleh	96	1.25	1.06
M2	Zahleh	97	/	/
M2	Zahleh	100	1.24	1.11
M2	Zahleh	135	1.24	1.07
M2	Zahleh	136	1.29	1.17
M2	Zahleh	137	1.21	1.14
M2	Zahleh	164	/	/
M3	Zahleh	16	0.73	0.84
M3	Zahleh	34	0.83	0.8
M3	Zahleh	40	/	0.88
M3	Zahleh	63	0.79	0.88
M3	Zahleh	69	0.86	0.9
M3	Zahleh	92	0.78	0.85
M3	Zahleh	108	/	/
M3	Zahleh	138	0.86	0.91
M3	Zahleh	168	0.84	0.89

m1	Zahleh	1	1.55	0.99
m1	Zahleh	8	1.63	1.04
m1	Zahleh	51	1.6	0.97
m1	Zahleh	79	/	/
m1	Zahleh	99	1.6	1.01
m1	Zahleh	139	1.56	0.97
m1	Zahleh	140	1.56	0.91
m2	Zahleh	44	1.18	0.96
m2	Zahleh	64	1.28	1.07
m2	Zahleh	65	1.13	0.99
m2	Zahleh	66	1.11	1.02
m2	Zahleh	72	/	0.96
m2	Zahleh	84	/	/
m2	Zahleh	89	1.22	1.08
m2	Zahleh	99	1.17	1.05
m2	Zahleh	128	1.16	1.01
m2	Zahleh	132	1.11	0.95
m2	Zahleh	133	1.18	1
m2	Zahleh	141	1.13	1.01
m2	Zahleh	142	1.15	1.03
m2	Zahleh	143	1.19	
m2	Zahleh	144	1.14	1.03
m2	Zahleh	145	/	/
m2	Zahleh	146	/	/
m2	Zahleh	147	/	/
m2	Zahleh	166	1.081	0.921
m2	Zahleh	167	1.024	0.852

m3	Zahleh	62	0.86	0.83
m3	Zahleh	67	0.92	0.91
m3	Zahleh	68	0.89	0.8
m3	Zahleh	71	0.88	0.77
m3	Zahleh	85	0.96	0.84
m3	Zahleh	86	0.96	0.87

Supplementary Table S2| Descriptive statistics of lower and upper molars of *Progonomys manolo* sp. nov. from Zahleh (Lebanon).

	**Length**					**Width**				
	N	Min.	Mean	Max.	SD	N	Min.	Mean	Max.	SD
m1	6	1.55	1.58	1.63	0.031	6	0.91	0.98	1.04	0.044
M1	7	1.69	1.78	1.90	0.073	9	1.05	1.14	1.26	0.066
m2	13	1.11	1.17	1.28	0.047	13	0.95	1.01	1.08	0.041
M2	11	1.18	1.25	1.33	0.049	11	1.03	1.13	1.23	0.069
m3	6	0.86	0.91	0.96	0.042	6	0.77	0.84	0.91	0.050
M3	7	0.73	0.81	0.86	0.048	8	0.80	0.87	0.91	0.036

Supplementary Table S3 | Sampling of the morphometric study, with documented taxa (genera, species), corresponding deposits, abbreviations used herein ("Codes"), numbers of first upper molars measured (Nb M1) and data sources.

Genus	Species	Deposit	Code	Nb M1	Reference
Progonomys	*manolo*	Zahleh (Bekaa Valley, central Lebanon)	ZAHLEH (PMAN-Z + Z-02)	9 (8+1)	This work
Antemus	*chinjiensis*	Locality 41 (Chinji Fm, Punjab, Pakistan)	ANTC	5	Kimura (unpublished data)
Castillomys	*crusafonti*	Sète (Hérault, France)	CC-STE	30	Deschamps[1]
Huerzelerimys	*minor*	Dionay (Isère, France)	HM-DIO	41	Renaud et al.[2]
	vireti	Lo Fournas 7 (Pyrénées-Orientales, France)	HV-LF7	3	Renaud et al.[2]
Karnimata	*darwini*	Locality 182A (Dhok Pathan Fm, Punjab, Pakistan)	KARD	6	Kimura (unpublished data)
	fejfari	Locality Y311 (Nagri Fm, Pakistan)	KARF	24	Plate from Kimura et al.[3]
	huxleyi	DP Locality 13 (Dhok Pathan Fm, Punjab, Pakistan)	KARH	6	Kimura (unpublished data)
Mus	*auctor*	DP Locality 13 (Dhok Pathan Fm, Punjab, Pakistan)	MUSAUC	7	Kimura (unpublished data)
Occitanomys	*sondaari*	Puente Minero (Teruel, Spain)	OS-PM	20	Renaud & van Dam[4]
		Tortajada A (Teruel, Spain)	OS-TOA	20	Renaud & van Dam[4]
Parapelomys	*robertsi*	DP Locality 13 (Dhok Pathan Fm, Punjab, Pakistan)	PPR	7	Kimura (unpublished data)
Parapodemus	*badgleyae*	Localities Y259, Y311 (Nagri Fm, northern Pakistan)	PABADG	4	Plate from Kimura et al.[3]
	lugdunensis	Dionay (Isère, France)	PAL-DIO	18	Renaud et al.[2]
	pasquierae	Lo Fournas 6 (Pyrénées-Orientales, France)	PAP-LF6	25	Renaud et al.[2]
Progonomys	*cathalai*	Montredon sup. (Hérault, France)	PRCT-MON	36	Renaud et al.[2]
		Soblay (Ain, France)	PRCT-SOB	38	Renaud et al.[2]
		Altintas (Kutahya, Turkey)	PRCT-ALT	11	Plate from Wessels[5]
	castilloae	Lo Fournas 7 (Pyrénées-Orientales, France)	PRCS-LF7	46	Renaud et al.[2]

	clauzoni	Dionay (Isère, France)	PRCL-DIO	24	Renaud et al.[2]
		Lo Fournas 6 (Pyrénées-Orientales, France)	PRCL-LF6	67	Renaud et al.[2]
	debruijni	Locality 182A (Dhok Pathan Fm, Punjab, Pakistan)	PRDEB	6	Kimura (unpublished data)
	hispanicus	La Roma 4B (Teruel, Spain)	PRH-R4B	4	Renaud & van Dam[4]
		La Roma 4C (Teruel, Spain)	PRH-R4C	8	Renaud & van Dam[4]
		Masia del Barbo 2B (Teruel, Spain)	PRH-MBB	20	Renaud & van Dam[4]
		Peralejos D (Teruel, Spain)	PRH-PERD	16	Renaud & van Dam[4]
		Dionay (Isère, France)	PRH-DIO	15	Renaud et al.[2]
		Pezinok (Bratislava region, Slovakia)	PRH-PEZ	2	Plate from Joniak[6]
	hussaini	Localities Y450, Y311, Y259 (Nagri Fm, Pakistan)	PRHUSS	10	Plate from Kimura et al.[3]
	minus	Locality 8A (Sinap Tepe, Turkey)	PRMIN	2	Plate from Sen[7]
	morganae	Locality Y450 (Hasnot area, Nagri Fm, Pakistan)	PRMORG	2	Plate from Kimura et al.[3]
	shalaensis	Shala (Nei Mongol, China)	PSHAL	1	Plate from Qiu and Li[8]
	sinensis	Localities 19, 38 (Shaanxi, China)	PRSIN	4	Plate from Qiu et al. [9]
	ibrahimi	Locality 84 of Sinap Tepe (Ankara, Turkey)	PRIBRA	4	Plate from Sen[7]

Supplementary Table S4 | Differences in length, width and length/width ratio between Zahleh (excluding Zahleh 02) and likely close relatives. P-values based on pairwise t-tests. In bold, significant probabilities.

		Length	Width	L/W
Z-rest	Early *P. hispanicus*	**0.0078**	**0.0115**	0.6239
	P. debruijni	0.5916	0.9870	0.4510
	P. hussaini	**< 0.0001**	**< 0.0001**	**0.0173**
	P. sinensis	0.7210	0.8010	0.8360

Supplementary Text S1|

Paratypes

Right M1 (Zahleh 15, Zahleh 127, Zahleh 95, Zahleh 134, Figs. 2B, D, E, G and Supplementary Figure S1); left M1 (Zahleh 94, Zahleh 90, Zahleh 83 Fig. 2H, J, K); right second upper molars (M2) (Zahleh 37, Zahleh 76, Zahleh 81, Zahleh 88, Zahleh 100, Zahleh 135, Zahleh 136, Fig. 2C, I and Supplementary Figure S1); left M2 (Zahleh 48, Zahleh 82, Zahleh 92, Zahleh 96, Zahleh 97, Zahleh 137 (broken) Fig. 2F, L, M and Supplementary Figure S2); right third upper molars (M3) (Zahleh 34, Zahleh 40, Zahleh 63, Zahleh 69, Zahleh 72, Zahleh 73, Zahleh 108, Fig. 2N-P and Supplementary Figure S1); left M3 (Zahleh 16, Fig. 2Q); right first lower molars (m1) (Zahleh 1, Zahleh 51, Zahleh 79, Zahleh 91, Zahleh 139, Zahleh 140, Fig. 3C-F), left m1 (Zahleh 8, Zahleh 8bis, Zahleh 125, Fig. 3A-B and Supplementary Figure S1); right second lower molars (m2) (Zahleh 44, Zahleh 64, Zahleh 65, Zahleh 66, Zahleh 72, Zahleh84, Zahleh 129, Zahleh 133, Zahleh 141, Zahleh 143, Zahleh 144, Zahleh 145 (only anterior part), Fig. 3G, H, J, K, L and Supplementary Figure S1); left m2 (Zahleh 89, Zahleh 99, Zahleh 128, Zahleh 132, Zahleh 141, Zahleh 142,

Zahleh 146 (only posterior part), Zahleh 147 (only anterior part) Fig. 3I, M); right third lower molars (m3) (Zahleh 62, Zahleh 68, Zahleh 85, Zahleh 86, Fig. 2N, O, P, R and Supplementary Figure S1); left m3 (Zahleh 71, Fig. 3Q).

Comparisons

With other murines (but *Progonomys*)

Progonomys manolo differs from the members of the tribe Arvicanthini in being much smaller, in having the occlusal outline of M1 much more elongated and with the cusps not aligned transversally and in having a well-developed posterior cingulum. It differs from the members of the tribe Murini in having a posterior cingulum on M1 and M2, a larger M3 with a well-developed t3 and in having rather symmetrical prelobe on m1. It can be distinguished from the members of the tribe Otomyini in having well-defined cusps and cingula on the cheek teeth, lacking the typical laminar pattern. It differs from the members of the tribe Malacomyini in having a well-defined t9 and a posterior cingulum on M1, well-defined cuspids not fused in chevrons on the lower molars and a well-developed C1 on m1. It differs from the members of the tribe Apodemyini in lacking any longitudinal connection between cusps, in the absence of cusp t7 and posterior spur on t3 on the upper molars, in lacking the medial anterior cuspid (tma) and in having the labial cingular cuspids less developed on the m1. It differs from the members of the tribe Rattini and Hydromyini in having the molars more cuspidate than laminate, in having a distinct posterior cingulum and well-developed labial cusps on the upper molars and labial cuspids on the lower molars.

Progonomys manolo from Lebanon clearly differs from all fossil rodents except those of its genus. Thus, it is different from species of *Potwarmus* in having cusp t1 on M1 and M2 and in having labial accessory cuspids and a double anteroconid on m1. It is distinct from

Antemus in having cusp t1 on M2 and cusps t4 and t5 and t4 and t8 connected by a ridge. It can be distinguished from *Karnimata* in having on M1 cusp t1 situated posterior to t2-t3 and t9 large and parallel to t6 in labial view and from *Paraethomys*, *Hansdebruijnia*, *Occitanomys*, *Castillomys*, *Castromys*, *Apodemus*, *Parapodemus*, *Stephanomys*, *Huerzelerimys*, *Yunomys* and *Leilaomys* in lacking any labial longitudinal connection between cusps. It is unlike *Linomys* in having cusp t1 and, to a lesser degree, cusp t4, posterior to cusps t2-t3 and t5-t6, respectively, and in lacking the tma on m1. It differs from *Pelomys* in having t1 and t4 posteriorly placed and a large t12 on m1.

With *Progonomys*

Comparison with* Progonomys hussaini *Cheema, Raza, Flynn, Rajpar et Tomida, 2000

Progonomys hussaini was coined on the basis on 57 isolated teeth from Locality JAL-101 of the Nagri Formation, Jalalpur area, Potwar Plateau, Punjab, Pakistan[9]. Its holotype (PMNH 5062, a left M1) is housed in the Pakistan Museum of Natural History, Pakistan. It is highly possible that this sample encompasses, in fact, more than one species[10,11]. Additional specimens have been found at Nagri Formation localities Y 311, Y 450 and Y 259 (Potwar Plateau, Pakistan), the age range of which is 10.1-10.5 Ma[11]. The Lebanese taxon has M1 with cusp t1 to some extent more strongly connected to t2 than in *Progonomys hussaini*, in which the two cusps are joined by a low and narrow ridge that leaves t1 in a rather isolated position. The m1 of *Progonomys manolo* show weaker longitudinal connections between their cuspids than those belonging to *Progonomys hussaini* and a more developed labial cingulum and more numerous cingulum cuspids than in the latter species. In addition, the posterior cingulum on the m2 of *Progonomys manolo* is usually smaller and cuspid-like, whereas it is usually longer and ridge-like on the m2 of *Progonomys hussaini*.

Comparison with Progonomys morganae Kimura, Flynn et Jacobs, 2017

This taxon has been erected on the basis of 13 isolated molars from the early late Miocene (10.2 Ma) Y450 locality, Nagri Formation, Pakistan[11]. The holotype (YGSP 33180) is a left M1 provisionally housed in the Peabody Museum of Archaeology and Ethnology, Harvard University (Cambridge, USA). This species is clearly smaller than the new *Progonomys* from Lebanon. Moreover, there are some morphological differences that prevent assignation of the Lebanese taxon to it. First, *Progonomys morganae* is lacking a deep inflection at t1, whereas the specimens of *Progonomys manolo* have it. Moreover, M1 of *Progonomys morganae* have a quite long and slender t12, which is short and cusp-like in *Progonomys manolo*. Besides, according to Kimura[11], *Progonomys morganae* has a t1that is small on M2 and even smaller on M3, whereas this cusp is quite large on M2 and M3 of the new Lebanese taxon. Moreover, the anteroconid cuspids on m1 are more closely appressed to each other in *Progonomys morganae* than in *Progonomys manolo*. In addition, m1 of *Progonomys morganae* have fewer and less developed cingulum cuspids. In fact, whereas *Progonomys morganae* bears a C1 (variable in size) and a minute C3 or C4, all m1 of the Lebanese taxon bear at least three cingulum cuspids: a large C1 and C4 and a less developed but distinct C3.

Comparison with _Progonomys sinensis_ Qiu, Zheng et Zhang, 2004

This species was named on the basis of 76 isolated molars from the early late Miocene (*circa* 10 Ma[12]) of the Bahe Formation, Shaanxi Province, China[9]. The holotype (V 13717), a left M1, is housed in in the Institute of Vertebrate Paleontology and Paleoanthropology in Beijing (China). Even though *Progonomys sinensis* is a primitive species of *Progonomys*, its size is quite large for a *Progonomys*; it is much larger than the new Lebanese taxon. Apart from the size, there are also striking morphological differences between *Progonomys sinensis* and *Progonomys manolo*. In fact, most of the m1 of *Progonomys sinensis* (8 out of 12) are characterized by having a tma on m1, whereas it is absent in all Lebanese specimens.

Besides, m1 show the labial anteroconid located slightly but noticeably posterior to the lingual one, which is not the case in *Progonomys manolo*. Moreover, most m1 of *Progonomys sinensis* (9 out of 11) have the anteroconid connected to the protoconid-metaconid complex by a low ridge that originates from the labial anteroconid. In contrast, on m1 of *Progonomys manolo* this link is lacking in all but the most heavily worn specimens, in which the connection comes out from the lingual anteroconid instead of from the lingual one. Finally, m1 and m2 show better-developed labial cingula (usually continuous) and less developed cingulum cuspids in *Progonomys sinensis* than in the Lebanese species. This is particularly evident on m1 of *Progonomys sinensis*, in which only cuspid C1 is developed and only in about half the specimens.

Comparison with *Progonomys minus* Sen, 2003

Progonomys minus was created on the basis of 13 isolated molars[7]. Its holotype (ST8A-53) is a left M1 from the Late Miocene Locality 8A (circa 9.9 Ma[13]) of Sinap Tepe, Ankara, Turkey. It is housed in the Natural History Museum of Ankara (Turkey). This taxon, just like *Progonomys manolo*, is characterized by its small size, but it differs morphologically from the Lebanese taxon. For instance, m1 of *Progonomys minus* is characterized by a distinct and continuous labial cingulum that bears only a small C1 and a barely distinct C4, whereas those of *Progonomys manolo* show an interrupted labial cingulum that bears at least three cingulum cuspids: a large C1 and C4 and a less developed but distinct C3. In the same way, m2 of *Progonomys minus* have less-developed cingulum cuspids (A1 and a small C1) than those of the new Lebanese taxon, all of which show distinct A1, C3 and C1. Moreover, the posterior chevron on the m2 of *Progonomys minus* is curved to a greater degree than in *Progonomys manolo*. Besides, the only recorded M3 of *Progonomys minus* has t1 labially connected to t5, whereas t1 is isolated in *Progonomys manolo*.

***Comparison with Progonomys debruijni* Jacobs, 1978**

This taxon was coined on the basis of 50 isolated cheek teeth[14]. The holotype (YGSP 7739) is a left M1 from locality Y182, Dhok Pathan Formation, Punjab, Pakistan[14], with an estimated age of 9.2 Ma (MN10)[15]. It is provisionally housed in the Peabody Museum of Archaeology and Ethnology, Harvard University (Cambridge, USA). Additional material has been found at the coeval locality Y367, Punjab, Pakistan[15], and at the *circa* 8.9 Ma locality of Ladhyani, Bilaspur, Himachal Pradesh, India[16]. Both *Progonomys manolo* and *Progonomys debruijni* are small sized and have the lingual cusps on M1 posteriorly placed. However, the molars of *Progonomys debruijni* are slightly smaller than those of *Progonomys manolo* and t1 is more slender, elongated and posteriorly placed than that observed in the Lebanese specimens. Moreover, m1 of *Progonomys debruijni* have the labial cingulum less developed and stronger longitudinal connections between the anteroconid, the metaconid and the protoconid than in the Lebanese taxon. The m2 of *Progonomys debruijni* show less cingulum cuspids than *Progonomys manolo*. In fact, whereas most specimens of *Progonomys debruijni* only have developed the cingulum cuspid C1, all m2 of *Progonomys* from Zahleh show two cingulum cuspids (C1 and C3) and a few of them as many as three.

***Comparison with Progonomys shalaensis* Qiu and Li, 2016**

This taxon was coined on the basis of a single M1 (V19924) from the Late Miocene (8.5-9.0 Ma[17]) locality of Shala, Sonid Youqi, Nei Mongol, China[8]. It is housed in the Institute of Vertebrate Paleontology and Paleoanthropology in Beijing (China). This M1 is smaller than those belonging to the new Lebanese species. According to the description provided by Qiu and Li[8], the M1 of *Progonomys shalaensis* is characterized by low cusps, whereas M1 of *Progonomys manolo* have them quite tall. Due to the scarcity of the material, we cannot compare these two species any further. Interestingly enough, the morphometric analysis

shows that the outline of the M1 from Nei Mongol would plot between *Progonomys sinensis* and *Parapodemus badgleyae*.

Comparison with *Progonomys hispanicus* Michaux, 1971

This species was named on the basis of numerous molars recorded from the Late Miocene of Masía del Barbo, Teruel, Spain[18]. Its holotype, MBB 885, is an upper molar housed in the palaeontological collections of Utrecht University (The Netherlands)[18]. M1 of the Lebanese taxon present an elongated outline that fits well with the most ancient populations of *Progonomys hispanicus* from La Roma 4C and La Roma 4B, Teruel, Spain (dated9.5 and 9.6 Ma, respectively[19]). The molars of *Progonomys manolo* are about the same size as those of *Progonomys hispanicus*. However, there are morphological differences that prevent assigning the Lebanese taxon to this species. M1 of *Progonomys hispanicus* show higher lingual cusps, which are less antero-posteriorly compressed than those of *Progonomys manolo*. Cusp t1 on M1 of *Progonomys hispanicus* is less posteriorly placed and t12 is less developed than in the Lebanese taxon. Cusps t8 and t9 seem to be more reduced on M3 of *Progonomys hispanicus* than in those of the Lebanese taxon. With respect to the lower molars, the anteroconid on m1 of *Progonomys hispanicus* is less anteriorly placed than in *Progonomys manolo*, in which there is not enough room for a tma to be grown. The lingual cuspids appear to be less developed in the Lebanese taxon than in *Progonomys hispanicus* and cingulum cuspid C1 is isolated in *Progonomys manolo*, whereas it seems to be connected to the hypoconid in *Progonomys hispanicus*. Cingulum cuspid C1 is less developed on m2 of the Lebanese taxon than in *Progonomys hispanicus*.

Comparison with *Progonomys woelferi* Bachmayer et Wilson, 1970

This species was named on the basis of numerous maxillae and mandibles from the Late Miocene (*circa* 8.75 Ma[22]) of Kohfidisch, Austria[23]. Its holotype (No. 1970/1395) is a

maxillary fragment with Ml-M2 housed in the collections of the Natural History Museum in Vienna (Austria). *Progonomys manolo* is clearly smaller than *Progonomys woelferi* and the outline of its M1 is much more elongated. Additional morphological characters allow distinguishing easily the two taxa. For instance, M1 have cusp t1 less elongated and less posteriorly placed in *Progonomys woelferi* than in *Progonomys manolo*. The upper molars of *Progonomys woelferi* are characterized by having cusps t6 and t9 connected or very close to one another, whereas they are clearly separated in the Lebanese taxon. Moreover, m1 of *Progonomys woelferi* have a distinct tma and a strong connection between the anteroconid and the metaconid-protoconid complex, which is lacking in all m1 of *Progonomys manolo*.

Comparison with *Progonomys clauzoni* Aguilar, Calvet et Michaux, 1986

Progonomys clauzoni has been coined on the basis of one hundred isolated molars and fragmentary maxillae and mandibles from the Late Miocene of Lo Fournas 6b, Pyrénées-Orientales, France[24]. The holotype of this species (FOU6b nº26) is a right maxillary fragment with M1-M2 that is housed at the paleontological collections of the University of Montpellier (France). *Progonomys manolo* differs from *Progonomys clauzoni* in being smaller and in having the first upper molars narrower. In fact, the outline of M1 of the new Lebanese taxon is more elongated than that of *Progonomys clauzoni*. Moreover, t2 of M1 appears to be more anteriorly located in *Progonomys clauzoni* than in *Progonomys manolo*. No M1 of *Progonomys manolo* display additional cusps such as t1 bis or t4 bis, which have been observed in some specimens of *Progonomys clauzoni*[25]. *Progonomys clauzoni* has t4 and t8 separated from one another, whereas a cingulum connects them in *Progonomys manolo*.

Comparison with *Progonomys castilloae* Aguilar et Michaux, 1996

This species was coined on the basis of one mandible with m1-m2, a maxilla with M1-M3, a maxillary fragment with M1 and numerous isolated teeth from the Late Miocene of Lo

Fournas 7, Pyrénées-Orientales, France[26]. Its holotype (FOU7 n°4) is a left m1 housed in the paleontological collections of the University of Montpellier (France). *Progonomys castilloae* differs from *Progonomys manolo* in being larger, in having t1 more rounded and less posteriorly situated on M1 and t4 usually not connected to t8. Moreover, m1 of *Progonomys castilloae* have the anteroconid connected to the metaconid and show less developed cingulum cuspids than the Lebanese taxon.

Comparison with *Progonomys ibrahimi* (Sen, 2003)

This species was coined by Sen[7] as the type of the new genus *Sinapodemus* on the basis of some dental characters such as the elongated outline of M1 with a well-marked indentation, elongated lingual cusps with t4 connected to t8, presence of a posterior spur on t6 and the absence of tma. In fact, except for the strong anteroconid-metaconid connection on m1, which is a character that recalls more evolved *Progonomys*, all the features described above are found in early populations of *Progonomys*. Therefore, the unassignment of this species to the genus *Progonomys* is, in our opinion, not justified. So, we consider *Sinapodemus* as a subjective junior synonym of *Progonomys* and *Progonomys ibrahimi* as a primitive representative of the genus.

The holotype of *Progonomys ibrahimi* (ST84-236) is a right M1 from the Late Miocene locality 84 (*circa* 9.4 Ma[13]) of Sinap Tepe, Ankara, Turkey[7]. It is housed in the Natural History Museum of Ankara (Turkey). This taxon, just like *Progonomys manolo*, is characterized by its small size, but it differs morphologically from the Lebanese taxon in having M1 with a more developed posterior spur on t6 and a strong connection between the lingual anteroconid and the metaconid on m1, which is absent on most m1 of the Lebanese taxon.

Comparison with *Progonomys cathalai* Schaub, 1938

Several populations of *Progonomys* have been attributed to *Progonomys cathalai*, but they probably belong to a different species. This is the case of the specimens from the oldest African localities of *Progonomys* such as Bou Hanifia 2 and 5 and Oued Zra, Algeria[27,28]. In fact, these specimens appear less derived than *Progonomys cathalai*, particularly in the far back position of t1 and the presence of a t9 that is clearly isolated from t6 on M1. Similarly, the *Progonomys* material described from the Turkish assemblages of Altıntaş and Kutahya and assigned to *Progonomys cathalai* by Wessels[5] is noticeably smaller (see supplementary figure S4) and morphologically different from the type population of *Progonomys cathalai* from Montredon. So, it is plausible that a revision of this material would provide evidence that it belongs, in fact, to a species different from, and more primitive than, *Progonomys cathalai*. In the same line of thought, the limited collection from level TU6/7 (circa 10.5-11 Ma) of the Tuḡlu Formation (Turkey), assigned by Joniak and de Bruijn [21] to *Progonomys cathalai*, may actually belong to an early species of *Progonomys* different from *Progonomys cathalai*.

Progonomys cathalai was coined on the basis of various isolated molars found in Montredon, Hérault, France[20]. Its holotype, A. Mo. 584, is a right M1 housed in the Naturhistorisches Museum Basel (Switzerland). *Progonomys manolo* is clearly smaller than *Progonomys cathalai* and the outline of its M1 is much more elongated. The new Lebanese taxon differs from *Progonomys cathalai* in having t1 more posteriorly situated with respect to t2-t3 and more anteroposteriorly compressed, and well-separated t6 and t9. Regarding the lower molars, *Progonomys manolo* has a weak and low connection between the lingual anteroconid and the metaconid, which is stronger in *Progonomys cathalai*. In addition, the cusps are more slender and tapering than in *Progonomys cathalai*. Moreover, the cheek teeth of *Progonomys*

manolo are noticeably smaller than those belonging to the type population of *Progonomys cathalai*.

Comparison with *Progonomys* from Africa

Progonomys has been only found in Northern Africa (Morocco, Algeria, Libya and, maybe, Egypt). Three species have been recorded therein: *Progonomys cathalai, Progonomys chougrani* and *Progonomys mauretanicus*. However, as seen above, several populations attributed to *Progonomys cathalai*[27,28] are less derived than *Progonomys cathalai* so much so that they probably belong to an unnamed species of *Progonomys* (*Progonomys* sp. 1) Concerning *Progonomys chougrani* and *Progonomys mauretanicus,* they have been described in unpublished doctoral theses[28,29], and are, therefore, unavailable till their formal publication. Moreover, according to Sen[7], *Progonomys chougrani* and *Progonomys mauretanicus* are identical to one another and likely represent the same species, which is very similar in morphology and size to *Progonomys cathalai*. We agree with Sen[7] in considering that the two species may actually be the same. However, the specimens on which they are based are noticeably larger than the specimens of *Progonomys cathalai* (see Supplementary Figure S4). So, they probably belong to a new, large species of *Progonomys* (*Progonomys* sp. 2).

Progonomys manolo differs from *Progonomys* sp. 1 in having the anteroconid and the protoconid-metaconid strongly connected. The labial cingula as well as the cingulum cuspids are stronger than in the Lebanese taxon and many m1 show a well-developed tma. One of the more striking differences between *Progonomys manolo* and *Progonomys* sp. 2 is that the latter taxon is much larger (see Supplementary Figure S4).

References

1. Deschamps L. *Dynamique et régulation de la diversité morphologique* (DEA dissertation, Université Lyon 1, 2004).
2. Renaud, S., Michaux, J., Mein, P., Aguilar, J.-P. & Auffray, J.-C. Patterns of size and shape differentiation during the evolutionary radiation of the European Miocene murine rodents. *Lethaia* **32**, 61–71 (1999).
3. Kimura, Y., Flynn, La. & Jacobs, L. Early Late Miocene Murine Rodents from the upper part of the Nagri Formation, Siwalik Group, Pakistan, with a new fossil calibration point for the Tribe Apodemurini (*Apodemus*/*Tokudaia*). *Fossil Imprint* **73**, 197–212 (2017).
4. Renaud, S. & van Dam, J. Influence of biotic and abiotic environment on dental size and shape evolution in a Late Miocene lineage of murine rodents (Teruel Basin, Spain). *Palaeogeogr. Palaeoclimatol. Palaeoecol.* **184**, 163–175 (2002).
5. Wessels, W. Miocene rodent evolution and migration: Muroidea from Pakistan, Turkey and Northern Africa. *Geol. Ultraiectina* **307**, 1–290 (2009).
6. Joniak, P. Upper Miocene rodents from Pezinok in the Danube Basin, Slovakia. *Acta Geol. Slov.* **8**, 1–14 (2016).
7. Sen, S. Muridae and gerbillidae (Rodentia). *Geology and Paleontology of the Miocene Sinap Formation* (eds Fortelius, M., Kappelman, J., Sen, S. & Bernor, R. L.) 125–140 (Columbia University Press, 2003).
8. Qiu, Z.-D. & Li, Q. Neogene rodents from central Nei Mongol, China [in Chinese with English summary]. *Palaeontol. Sin. C* 30: i–xi, 1–684 (2016).
9. Qiu, Z.-D., Zheng, S.-H. & Zhang, Z.-Q. 2004. Murids from the Late Miocene Bahe Formation, Lantian, Shaanxi. *Vertebr. PalAsiat.* **42**, 67–76.
10. Cheema et al. Miocene Small Mammals from Jalalpur,P kistan, Biochronologic Implications. *Bull. Nat. Sci. Mus. TokyoC* **26**, 57–77 (2000).
11. Kimura, Y., Flynn, La. & Jacobs, L. Early Late Miocene Murine Rodents from the upper part of the Nagri Formation, Siwalik Group, Pakistan, with a new fossil calibration point for the Tribe Apodemurini (*Apodemus*/*Tokudaia*). *Fossil Imprint* **73**, 197–212 (2017).
12. Zhang, Z.-Q. *et al.* Mammalian Biochronology of the Late Miocene Bahe Formation. *Fossil Mammals of Asia: Neogene Biostratigraphy and Chronology* (eds Wang, X., Flynn L. J. & Fortelius, M.) 187–201 (Columbia University Press, 2013).

13. Kappelman *et al*. *Chronology, Geology and Paleontology of the Miocene Sinap Formation* (eds Fortelius, M., Kappelman, J., Sen, S. & Bernor, R. L.) 41–66 (Columbia University Press, 2003).

14. Jacobs, L. L. Fossil rodents (Rhizomyidae and Muridae) from Neogene Siwalik deposits, Pakistan. *Mus. North. Ariz. Bull.* **52**, 1–103 (1978).

15. Kimura, Y. Hawkins, M. T. R., McDonough, M. M., Jacobs, L. L. & Flynn, L. J. Corrected placement of *Mus-Rattus* fossil calibration forces precision in the molecular tree of rodents. *Sci. Rep.* **5**, 14444 (2015).

16. Patnaik, R. Indian Neogene Siwalik Mammalian Biostratigraphy. *Fossil Mammals of Asia: Neogene Biostratigraphy and Chronology* (eds Wang, X., Flynn L. J. & Fortelius, M.) 423–444 (Columbia University, 2013).

17. Wang, X., Flynn L. J. & Fortelius, M. Toward a Continental Asian Biostratigraphic and Geochronologic Framework. *Fossil Mammals of Asia: Neogene Biostratigraphy and Chronology* (eds Wang, X., Flynn L. J. & Fortelius, M.) 1–25 (Columbia University Press, 2013).

18. Michaux, J. Muridae (Rodentia) Neogenes d’Europe Sud-Occidentale. Evolution et rapports avec les formes actuelles. *Paleobiol. Cont.* **1**, 1–67 (1971).

19. Van Dam, J. The small mammals from the Upper Miocene of the Teruel-Alfambra region (Spain): paleobiology and palaeoclimatic reconstructions. *Geol. Ultraiectina* **156**, 1–290 (1997).

20. Schaub, S. Tertiäre und quartäre Murinae. *Schweiz. Palaeontol. Abh.* **61**, 1–38 (1938).

21. Joniak, P. & de Bruijn, H. Rodents from the Upper Miocene Tuğlu Formation (Çankırı Basin, Central Anatolia, Turkey). *Paläontol. Zeit.* **89**, 1039–1056 (2015).

22. Böhme, M., Ilg, A. & Winklhofer, M. Late Miocene “washhouse” climate in Europe *Earth Planet. Sci. Lett.* **275**, 393–401(2008).

23. Bachmayer, F. & Wilson, R.W. Die Fauna der altpliozänen Höhlen- und Spaltenfüllungen bei Kohfidisch, Burgenland (Österreich). *Ann. Naturhistor. Mus. Wien* **74**, 533–587 (1970).

24. Aguilar, J.-P., Calvet, M. & Michaux, J. Découvertes de faunes de micromammifères dans les Pyrénées-Orientales (France) de l’Oligocène supérieur au Miocène supérieur ; espèces nouvelles et réflexion sur l’étalonnage des échelles continentale et marine. *C.R. Acad. Sci. Paris* II, **303**, 755–760 (1986).

25. Lazzari, V., Aguilar, J.-P. & Michaux, J. Intraspecific variation and micro-macroevolution connection: illustration with the late Miocene genus *Progonomys* (Rodentia, Muridae). *Paleobiology*, **36**, 641–657 (2010).

26. Aguilar, J.-P. & Michaux, J. The beginning of the age of Murinae (Mammalia: Rodentia) in southern France. *Acta Zool. Cracov.* **39**, 35–45 (1996).

27. Jaeger, J.-J. 1977. Les Rongeurs du Miocene moyen et supérieur du Maghreb. *Palaeovertebrata* **8**, 1–166.

28. Ameur-Chebbeur, A. *Biochronologie des Formations continentales du Néogène et du Quaternaire de l'Algérie : Contribution des Micromammifères* (PhD dissertation, Université d'Oran, 1988).

29. Coiffait-Martin, B. *Contribution des rongeurs du Néogène d'Algérie à la biochronologie mammalienne d'Afrique Nord-Occidental* (PhD dissertation, Université de Nancy, 1991).
